# Exploring Evaluation of eHealth Lifestyle Interventions for Preschool Children: A Scoping Review

**DOI:** 10.1016/j.mcpdig.2025.100223

**Published:** 2025-04-17

**Authors:** Marissa C.J. Kooij, Ashley J.P. Smit, Linda D. Breeman, Lieke Schiphof-Godart, Isra Al-Dhahir, Andrea W.M. Evers, Koen F.M. Joosten

**Affiliations:** a Department of Neonatal and Pediatric Intensive Care, Erasmus MC Sophia Children’s Hospital, Rotterdam, Netherlands; b Department of Medical Informatics, Erasmus MC Sophia Children’s Hospital, Rotterdam, Netherlands; c Health, Medical, and Neuropsychology Unit, Leiden University, Leiden, Netherlands

## Abstract

EHealth lifestyle interventions can promote positive lifestyle changes in preschool children, but they need to be evaluated to assess their effectiveness and identify areas for improvement. This scoping review aimed to examine evaluation methods, outcome measures, and methodologic strengths and weaknesses, to provide recommendations for the evaluation of eHealth lifestyle interventions for preschool children. A comprehensive literature search was conducted across 6 databases for articles published up to September 29, 2023. We identified 48 articles describing 31 interventions that met our predefined eligibility criteria. These interventions predominantly targeted children’s diet. The most frequently evaluated outcomes were effectiveness, acceptability, and usage. Effectiveness outcomes included, among others, dietary intake, anthropometrics, and child and parental behaviors. Acceptability was evaluated primarily as user satisfaction. Evaluation methods for effectiveness and acceptability included questionnaires, interviews, focus groups, and portable devices. Intervention usage was evaluated via logged use and self-reported data. On the basis of our findings, we present recommendations for future evaluation of eHealth interventions for preschool children. These recommendations focus on selecting relevant outcome measures and appropriate evaluation methods and on integrating and applying evaluation results.

**Article Highlights**

• This article reviews 48 studies on 31 eHealth interventions targeting preschoolers’ lifestyles at various development stages.
• Most studies focused on short-term diet-related outcomes, with promising results, but long-term effects—especially on physical activity and sleep—remain unclear. Assessing outcomes like usability, acceptability, cost-effectiveness, and adoption are crucial for understanding long-term success and real-world impact.
• We recommend the use of standardized definitions and frameworks for key outcomes to improve consistency and comparability.
• We advocate the use of objective measures to reduce bias and detailed usage data to better understand adherence for future improvements.

Preschool children often do not comply with healthy lifestyle recommendations.^1^ This could potentially lead to myopia, tooth decay, and delayed motor and cognitive development.^2^, ^3^, ^4^, ^5^ Moreover, an unfavorable lifestyle characterized by high intake of sugar-sweetened beverages, insufficient physical activity, a high prevalence of sedentary behavior, and inadequate sleep is associated with adverse health outcomes, with overweight being the most notable.^5^, ^6^, ^7^, ^8^ In 2023, 5.6% of children under 5 years worldwide were overweight.^9^ Early childhood is a critical period where lifestyle patterns are established, often persisting into later life.^10^^,^^11^ Obese children are more likely to be obese and face negative cardiovascular outcomes in adulthood compared with nonobese children.^12^^,^^13^ These findings underscore the importance of addressing unhealthy behaviors early in life.

In response to this challenge, eHealth lifestyle interventions have emerged as promising tools. Digital solutions can assist parents in promoting healthy behaviors in children and changing unhealthy ones.^14^ EHealth interventions offer numerous benefits compared with regular health care, including enhanced anonymity, constant accessibility, and scalable outreach to the target audience.^15^ Many interventions targeting the lifestyle of preschool children show promise in increasing physical activity and reducing sedentary behavior.^16^ However, effectiveness should not be the only outcome of interest. Other outcomes, such as acceptability and usage, are important to understand why the technology was successful or not and provide insights for enhancing the design, implementation, adoption, and use of eHealth interventions.^15^^,^^17^ For example, Karssen et al^18^ found that the initial effects of their eHealth intervention on child body mass index (BMI) faded after 6 months and disappeared by 12 months. A potential explanation was a lack of sustained application use after 6 months,^18^ highlighting the need for strategies to encourage sustained usage. In addition, evaluating reach, cost-effectiveness, and engagement is important for understanding the broader impact and real-world applications of these interventions.^19^ When evaluating eHealth lifestyle interventions, it is also important to realize that the chosen outcome measure and evaluation method markedly influence the results and conclusions, owing to inherent biases in each evaluation method.^20^ In addition, it is important to select outcome measures that are sensitive to the degree of expected change.^20^

### This Study

Evaluating eHealth interventions can guide future iterations through a better understanding of how they are effective, for whom to create, and underlying reasons behind their effectiveness.^15^ However, there are different outcome measures and methods to evaluate eHealth lifestyle interventions for preschool children. Moreover, few reviews about eHealth lifestyle interventions for children explore outcomes beyond effectiveness.^19^ Therefore, this scoping review provides an overview of evaluated outcome measures and corresponding evaluation methods of eHealth lifestyle interventions for preschool children, and their strengths and limitations. We also report on the results of these evaluations. In addition, we provide recommendations for the future evaluation of eHealth interventions targeting preschool children.

## Methods

### Design: Scoping Review

A scoping review was chosen as the most appropriate method to summarize and synthesize research findings on the evaluation methods of eHealth interventions aimed at changing the lifestyle of preschool children. We followed the 2018 Preferred Reporting Items for Systematic Reviews and Meta-Analyses Extension for Scoping Review (PRISMA-ScR) checklist^21^ and the methodologic framework by Arksey and O’Malley for scoping reviews.^22^ A review protocol was not published.

### Search Strategy

A medical librarian from Erasmus Medical Centre developed a search strategy along with 2 authors (A.J.P.S. and M.C.J.K.), which included the key terms for preschool-aged children, digital interventions, and lifestyle components. The full search strategy is available in Supplemental Appendix 1 (available online at https://www.mcpdigitalhealth.org/). Databases searched included medline ALL (1946-2023), Embase (1971-2023), Web of Science (1975-2023, CINAHL (1982-2023), and Google Scholar. Duplicate findings were removed. In addition, the reference lists of the included articles after full-text screening were searched to identify additional relevant articles. The databases were searched articles published up to September 29, 2023.

### Eligibility Criteria and Screening

The screening of titles and abstracts was realized using ASReview (v1.1), a machine-assisted open-source screening tool. The ASReview algorithm identifies relevant articles based on the articles that are included or excluded by the reviewer. The program orders articles based on relevance and presents the most relevant articles first. The predefined stopping rules for screening entailed screening a minimum of 36.1% of the articles and encountering 25 consecutive nonrelevant articles.^23^ First, 2 authors (A.J.P.S. and M.C.J.K.) independently screened the title and abstract for eligibility using the inclusion and exclusion criteria defined in Table 1.^24^ Articles were also included for full-text screening when no abstract was available. Second, both authors (A.J.P.S. and M.C.J.K.) performed a full-text screening of the included articles to check against the inclusion and exclusion criteria. Discrepancies were discussed with the other coauthors.

**Table 1:** Inclusion and Exclusion Criteria

Inclusion criteria
Articles were included if they: • described an eHealth (applications, websites, social media, and SMS) intervention; • targeted the lifestyle of preschool children (0-6 years old); • focused on at least 1 of the following lifestyle components: physical activity/sedentary behavior, diet, sleep, or screen time; • presented information on evaluation of the intervention and the results of this evaluation; • took place in a high-income country^a^; • had a full-text available in English.

Exclusion criteria
Articles were excluded when they: • targeted medically vulnerable children or parents (eg, with diabetes); • targeted health professionals or teachers/daycare workers; • primarily targeted the lifestyle of the parents or caregivers of preschool children; • focused primarily on breast or formula feeding; • comprised <50% of eHealth components; • were a telemedicine intervention, an online advertisement, or campaign; • were case reports or reviews.

a According to the World Economic situation and prospects 2023 report by the United Nations.^24^

### Data Extraction and Synthesis

Study characteristics were independently extracted by both authors (A.J.P.S. and M.C.J.K.) (Supplemental Appendix 2, available online at https://www.mcpdigitalhealth.org/). The various stages of development of the included studies were identified in accordance with the WHO guide *Monitoring and Evaluating Digital Health Interventions*.^25^ In addition, all evaluated outcome measures were extracted (eg, number of fruits eaten and minutes spent using the intervention) and categorized according to predefined outcome definitions (Supplemental Appendix 3, available online at https://www.mcpdigitalhealth.org/). These definitions served as guidelines for data synthesis. In case of a discrepancy between our predefined definition and the authors’ terminology, the evaluated outcome measure was categorized according to our predefined definition to ensure a homogeneous analysis. Engagement was defined as a construct encompassing affective, cognitive, and behavioral user experience. However, these components overlap with those found in the definitions of usage, usability, and acceptability. Therefore, we did not include engagement as a separate outcome in our review. In addition, several researchers evaluated constructs that can act as mediators on the pathway toward behavior change (eg, knowledge and attitudes), often based on behavior change theories.^26^, ^27^, ^28^, ^29^, ^30^, ^31^, ^32^, ^33^, ^34^, ^35^, ^36^ These mediators were not examined in this review.

For each outcome measure, the evaluation method and results were extracted. Long-term evaluation was defined as evaluating the intervention at least 6 months after the most recent intervention interaction.^37^ Furthermore, the strengths and limitations of the evaluation method as described by the authors were collected. The results are presented per outcome. Effectiveness, acceptability, and usage were most frequently evaluated and are therefore discussed in more detail. Their definitions are provided in Supplemental Appendix 3.

## Results

### Study Selection

**Figure 1:**
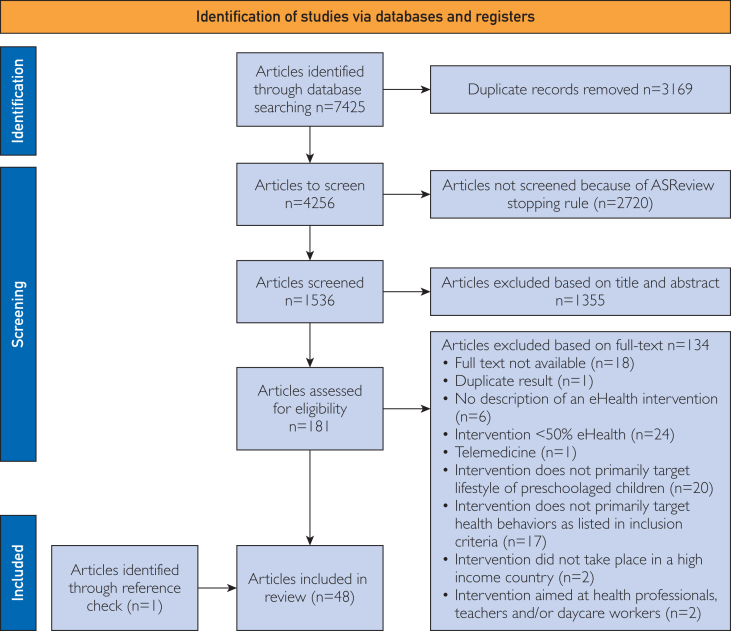
Flowchart depicting article screening process.

The systematic search across the databases revealed 4256 potentially relevant citations. After screening 1536 titles and abstracts with ASReview, 181 articles were included for full-text screening. Of these articles, 47 met all inclusion criteria and 1 article was identified through reference checking, resulting in 48 included articles (Figure 1).

### Description of the Included Studies

A summary of the characteristics of the included articles is presented in Table 2.^18^^,^^25^, ^26^, ^27^, ^28^, ^29^, ^30^, ^31^, ^32^, ^33^, ^34^, ^35^, ^36^^,^^38^, ^39^, ^40^, ^41^, ^42^, ^43^, ^44^, ^45^, ^46^, ^47^, ^48^, ^49^, ^50^, ^51^, ^52^, ^53^, ^54^, ^55^, ^56^, ^57^, ^58^, ^59^, ^60^, ^61^, ^62^, ^63^, ^64^, ^65^, ^66^, ^67^, ^68^, ^69^, ^70^, ^71^, ^72^, ^73^ A complete overview can be found in Supplemental Appendix 2. The 48 included articles describe 31 different interventions. The eHealth modalities most used by these interventions were applications and websites (both n=11), of which 2 interventions later added a Facebook page. Many interventions targeted multiple lifestyle components (n=16). Diet was the most targeted lifestyle component (n=24). All interventions were designed for use by parents or caregivers, except for the intervention “Jungle gym,”^57^ which targeted preschool children directly. Furthermore, the Fanmeal application offered both a separate parental mode and a serious game mode designed for preschool children.^34^^,^^48^

**Table 2:** Summary of Intervention Characteristics

Study name	eHealth modality	Targeted lifestyle component	Targeted population	Duration of intervention	First author, year, phase(s) of testing^a^
Babysleep	Website	Sleep	Parents of children aged 0 to 3 y	NA	Mindell et al,^38^ 2021, preprototype and scale-up
Bedtime Routines Intervention for Children (BRIC)	SMS	Sleep	First time parents of children aged 1-3 y	7 d	Kitsaras et al,^39^ 2022, preprototype, prototype, and pilot
Children Eating Well	Application	Diet	Families with preschool children aged 2-4 y, participating in WIC	3 mo	Hull et al,^40^ 2017, preprototype and prototype
Cooking matters	Application	Diet	Low-income caregivers of children aged 5 y or younger	2 mo	Garvin et al,^41^ 2019, pilot
Cooking matters	Facebook	Diet	Low-income families (from pregnancy up to children aged 5 y)	NA (current users were asked for participation in surveys and interviews)	Zhang et al,^28^ 2021, pilot
Creating Healthy Habits Among Maryland Preschoolers (CHAMP)	Website	Diet and physical activity	Parents of children aged 3-5 y	The intervention lasted 29 or 33 wk	Ezran et al,^42^ 2021, pilot
The Customzed Sleep Profiel (CSP)	Website	Sleep	Mothers of children aged 6-36 mo with a parent-identified sleep problem	3 wk	Mindell et al,^43^^,^^44^ 2011, pilot
Early Food for Future Health	Website	Diet	Parents of infants aged 3-5 mo	7 mo	Helle et al,^45^^,^^46^ 2019, pilot
Enabling Mothers to Prevent Pediatric Obesity Through Web-Based Education and Reciprocal Determinism (EMPOWER)	Website	Diet, physical activity, and screen time	Mothers of children aged 4 to 6 y	4 wk, with a booster session delivered within the 4 wk after this	Knowlden and Sharma,^47^ 2014, pilot Knowlden et al,^31^ 2015, pilot Knowlden and Sharma,^30^ 2016, pilot Knowlden and Conrad,^32^ 2018, pilot
Fanmeal	Application	Diet, physical activity, and sleep	Parents of children aged 3-6 y	4 wk	Afonso et al,^34^^,^^48^ 2020, pilot and prototype and pilot, respectively
Food4toddlers	Website	Diet	Parents of children aged 1 y	6 mo	Røed et al,^49^ 2020, pilot Røed et al,^50^ 2021, pilot
Grow2Gether	Facebook	Diet, physical activity, and sleep	Mothers of children aged 0-12 mo, who were obese before pregnancy	8 wk	Gruver et al,^35^ 2016, preprototype and pilot
Grow2Gether	Facebook	Diet, sleep, and screen time	Mothers of infants aged 0-9 mo, at high risk of obesity	11 mo (2 mo prenatal)	Fiks et al,^51^ 2017, pilot
Head Start	Facebook	Diet	Caregivers of children aged 3-5 y, from Head Start families	3 wk	Lawton et al,^52^ 2022, pilot
Healthy Beginnings	SMS	Diet and physical activity	Low-income parents of children aged 0-5 y	12 wk	Evans et al,^29^ 2022, pilot
HEalthy EnviROnments (HEROS)	Application	Diet and physical activity	Mothers of children aged 3-5 y, enrolled in Head Start centers	6 wk	Reyes et al,^53^ 2023, pilot
Healthy Families, Healthy Kids 2–5 (HFHK2–5)	Website	Diet, physical activity, and screen time	Caregivers of overweight preschoolers aged 2-5 y	NA	Davies et al,^54^ 2014, preprototype, prototype, and pilot
Johnson’s Bedtime Baby Sleep Application	Application	Sleep	Caregivers of infants	NA (although there needed to be a minimum of 4 d and a maximum of 28 d between the first use [initial assessment] and second use of the CSP)	Leichman et al,^55^ 2020, demonstration
Jump2Health	Website	Diet, physical activity, sleep, and screen time	Parents of preschool children aged 3-5 y	NA	Taylor et al,^56^ 2016, pilot
Jungle gym	Application	Physical activity	Preschool children aged 3-5 y	Children tested the application once, lasting about 10-15 min per group	McCloskey et al,^57^ 2018, preprototype, prototype and pilot
Lessonly, Inc (Baby-Act Trial)	Website	Diet, physical activity, and sleep	Mothers from pregnancy to infants aged 1 y	NA	Kallis et al,^58^ 2023, pilot
Mini-KiSS Online	Website	Sleep	Parents of children aged 6 mo to 4 y	6 wk	Schlarb and Brandhorst,^59^ 2012, pilot
MINISTOP	Application	Diet, and physical activity	Parents of children aged 4 y	6 mo	Nystrom et al,^60^ 2017, pilot Nystrom et al,^61^ 2018, pilot
MINISTOP 2.0	Application	Diet, physical activity, and screen time	Parents of children aged 2.5-3 y	6 mo	Alexandrou et al,^62^ 2023, demonstration
Nenne Navi	Application	Sleep	Caregivers of infants aged 18 mo to 3 y	2 mo; 1 y	Yoshizaki et al,^63^ 2020, prototype Yoshizaki et al,^64^ 2023, pilot
Samen Happie!	Application	Diet, physical activity, and sleep	Parents of children aged 5-15 mo	1 y	Karssen et al,^18^ 2022, pilot
The Short Messaging System (SMS) Parent Action Intervention	SMS	Diet, physical activity, sleep, and screen time	Caregivers of children aged 3-5 y	5 wk	Brown et al,^33^ 2019, pilot
Skoolbag/SWAP IT	Application	Diet	Caregivers of children aged 3-6 y	10 wk	Pearson et al,^65^ 2022, pilot
Smartmoms	Website and SMS	Diet	Mothers with a high BMI, of children aged 3-5 y	6 mo	Nezami et al,^66^ 2018, pilot Nezami et al,^67^ 2020, pilot
Sugar fact intervention	YouTube videos	Diet	Mothers of children aged 1-6 y	2 × 15 min	Chen et al,^26^ 2020, pilot
Time2bHealthy	Website	Diet, physical activity, and screen time	Caregivers of children aged 2-5 y who were (at risk of being) overweight	10 wk	Jones et al,^36^ 2011, pilot
Time2bHealthy	Website and Facebook	Diet, physical activity, screen time, and sleep	Caregivers of children aged 2-5 y whose BMI was at or above the 50th percentile	11 wk (after which participants received e-mails until 6-mo follow-up)	Hammersley et al,^68^^,^^69^ 2019, pilot Hammersley et al,^70^ 2020, pilot
Time2bHealthy	Website and Facebook	Diet, physical activity, screen time, and sleep	Parents of children aged 2-6 y	12 wk (up to 20 wk to allow participants to complete the intervention)	Hammersley et al,^71^ 2021, demonstration Hammersley et al,^72^ 2022, demonstration
NA	Website and SMS	Diet, physical activity, and screen time	Parents of children aged 1-3 y	8 wk	Lee et al,^27^ 2023, pilot
NA	Tablet based	Diet, physical activity, and screen time	Mothers, identifying as Chinese, of children aged 3-5 y	8 wk	Sun et al,^73^ 2017, pilot

NA, not applicable; RCT, randomized controlled trial; WIC, woman, infants and children.

a In accordance with the WHO guide for monitoring and evaluating digital health interventions.^25^

**Figure 2:**
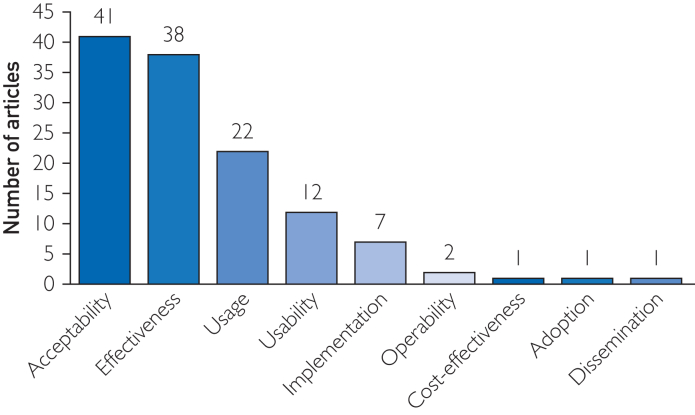
Bar chart representing the number of articles per evaluated outcome.

The studies described various phases of testing, including preprototype (n=6), prototype (n=10), pilot (n=40), demonstration (n=4), and scale-up (n=1). Most of the included articles evaluated the acceptability (n=41) and effectiveness (n=38) of their intervention, followed by usage (n=22) (Figure 2).

### Effectiveness

#### Outcome Measures and Evaluation Methods

Quantitative outcome measures used to assess effectiveness included parameters of sleep (eg, sleep duration), dietary intake (eg, amount of fruits and vegetables per day), anthropometrics (eg, BMI), screen time (eg, minutes of screen time per day), and physical activity (eg, time spent in moderate to vigorous physical activity per day). Behavior was also assessed, such as children’s sleep hygiene and their willingness to try new foods. Furthermore, some studies included parental outcomes (eg, attempted or actual behavior change). Questionnaires and interviews were the main methods for evaluating the intervention’s effectiveness. However, effectiveness was also measured with devices (eg, an actigraphy),^60^^,^^63^^,^^68^, ^69^, ^70^ with a diet diary,^70^ and from food pictures sent by parents.^60^^,^^61^ Supplemental Table 1 (available online at https://www.mcpdigitalhealth.org/) summarizes the outcome measures and methods used to evaluate the interventions’ effectiveness and example questions, grouped per lifestyle component.

#### Effectiveness Results

In most articles (n=34), researchers evaluated short-term effectiveness (measured within 6 months from the last day of the intervention). In the 13 randomized controlled trials (RCTs) evaluating short-term effectiveness, outcome measures significantly improved for sleep,^43^ diet,^27^^,^^31^^,^^46^^,^^51^^,^^60^^,^^62^^,^^66^^,^^67^^,^^69^^,^^73^ and screen time^27^^,^^62^ in the intervention group. Some studies found no significant improvement for 1 or more outcome measures, such as for sleep,^51^^,^^69^ diet,^28^^,^^65^ screen time,^31^^,^^51^^,^^69^ and physical activity^27^^,^^31^^,^^60^^,^^62^^,^^69^^,^^73^ in the intervention group. In a few RCTs (n=7), researchers (additionally) evaluated long-term effectiveness. Some reported significant long-term positive effects for sleep^44^ and specific diet outcome measures.^30^^,^^32^ Others were not able to show significant long-term results of their intervention for specific diet outcome measures,^30^^,^^45^^,^^50^^,^^61^^,^^71^ physical activity,^30^^,^^32^^,^^61^ and screen time.^30^^,^^32^^,^^71^ In multiple studies, children’s BMI was evaluated,^18^^,^^45^^,^^46^^,^^62^^,^^66^^,^^69^^,^^73^ but most did not find significant improvements in their intervention groups. However, in 1 study, researchers found an effect in BMI after 6 months, although this effect disappeared at the 12-month follow-up.^18^

### Acceptability

#### Outcome Measures and Evaluation Methods

In this review, acceptability was analyzed as a multifaceted construct (Supplemental Appendix 3). Most acceptability outcome measures in the included studies were related to users’ feelings (affective attitudes) toward the intervention.^4^, ^5^, ^6^, ^7^, ^8^, ^9^, ^10^, ^11^, ^12^, ^13^, ^14^, ^15^, ^16^, ^17^, ^18^, ^19^, ^20^, ^21^, ^22^, ^23^, ^24^, ^25^, ^26^, ^27^, ^28^, ^29^, ^30^, ^31^, ^32^, ^33^, ^34^, ^35^, ^36^, ^37^, ^38^, ^39^, ^40^, ^41^, ^42^, ^43^, ^44^, ^45^, ^46^, ^47^, ^48^^,^^51^, ^52^, ^53^, ^54^^,^^56^, ^57^, ^58^, ^59^^,^^62^, ^63^, ^64^, ^65^, ^66^^,^^69^^,^^72^^,^^73^ For example, Jones et al^36^ asked their participants about the interestingness, comprehensiveness, and relevance of their intervention’s content. Some studies explored the satisfaction of a specific intervention feature, such as an activity registration function,^62^ or the Facebook page that was added to a website.^70^ Additionally, participants were frequently asked about the likelihood of continued use and recommendation of the intervention to others.^18^^,^^29^^,^^35^^,^^51^, ^52^, ^53^^,^^58^^,^^65^ Self-efficacy,^27^^,^^30^, ^31^, ^32^^,^^41^^,^^49^^,^^55^^,^^59^^,^^62^^,^^67^^,^^68^ perceived intervention effectiveness,^29^^,^^33^^,^^35^^,^^36^^,^^43^^,^^45^^,^^48^^,^^49^^,^^51^, ^52^, ^53^^,^^55^^,^^59^^,^^65^^,^^69^^,^^70^^,^^72^^,^^73^ and the perceived amount of effort required to participate in the intervention^18^^,^^27^^,^^33^^,^^36^^,^^47^^,^^48^^,^^60^^,^^64^^,^^69^^,^^71^^,^^72^ were also assessed. The latter was done, for example, by asking about the acceptability of the intervention duration and reasons for attrition. Supplemental Table 2 (available online at https://www.mcpdigitalhealth.org/) summarizes the outcome measures to evaluate the interventions’ acceptability. Questionnaires were the predominant method for evaluating the acceptability of the interventions. In most studies, acceptability questionnaires were self-made, although, in some, it was mentioned that questions were used or derived from the Mobile Application Rating Scale, a tool for classifying and assessing the quality of mobile health applications.^18^^,^^53^

#### Acceptability Results

Overall, participants found the interventions acceptable. In 5 of the 6 studies assessing the acceptability of their intervention’s duration (1-3 months), most participants were satisfied.^33^^,^^36^^,^^48^^,^^69^^,^^72^ In a year-long intervention, about half of the participants found the duration appropriate, whereas 42% preferred a shorter, 6-month duration.^64^ Thirty-two studies reported attrition or retention rates, with attrition rates ranging from 0% to 79%. Reasons for dropout included sufficient perceived improvement of the targeted lifestyle behavior,^64^ internet connectivity problems,^36^^,^^64^ a lack of time and scheduling difficulties,^27^^,^^47^^,^^60^^,^^71^ a lack of interest,^18^^,^^71^ refusal to wear tracking devices,^60^ malfunctioning devices or intervention,^18^^,^^47^ personal reasons (eg, illness),^27^^,^^36^^,^^60^ needing to free up phone storage or changing phones,^18^ not receiving the download instructions or forgetting to download the intervention,^18^ and difficulties remembering intervention log-in data.^48^

### Usage

#### Outcome Measures and Evaluation Methods

Supplemental Table 3 (available online at https://www.mcpdigitalhealth.org/) summarizes the outcome measures and methods used to evaluate data on the user’s interactions with the intervention. Outcome measures were stratified by both parental self-reported use and logged use across all modalities. Although parental logged data occasionally provided detailed information on overall intervention use and the use of specific features, parental self-reported usage not only offered basic insights into usage but also revealed valuable information about the motivations behind use of the intervention and reasons for nonusage.^40^

Logged use was measured with usage logs^18^^,^^29^^,^^33^^,^^34^^,^^38^^,^^40^^,^^42^^,^^48^, ^49^, ^50^^,^^53^^,^^60^^,^^62^ and modality specific methods.^35^^,^^51^^,^^66^^,^^70^ For example, for a Facebook intervention, the number of reactions to Facebook posts were counted.^70^ Children’s usage was evaluated by observing their interaction with the application and reviewing video recordings of these interactions.^57^ Self-reported data were predominantly collected through self-made questionnaires and interviews.^18^^,^^28^^,^^35^^,^^40^^,^^41^^,^^46^^,^^49^^,^^51^^,^^58^^,^^62^

#### Usage Results

Overall, parental logged use results (n=17) were reported to be high. Three interventions found that parents watched all recommended videos and, in most cases, even the additional videos.^34^^,^^35^^,^^48^^,^^53^ Additionally, 77.7% of the participants read the recommended challenges, aimed at improving their knowledge and skills.^48^ High usage was also observed in an intervention that allowed responses to text messages, with 95% of participants responding.^33^ However, in some studies, the level of interaction varied per participant.^18^^,^^33^^,^^40^^,^^48^, ^49^, ^50^^,^^52^^,^^60^^,^^70^ For example, 1 study found that 44% of participants had 20 or more interactions with Facebook posts, whereas 28% had between 0 and 8 interactions.^52^ In addition, some studies found a decrease in usage over time^18^^,^^48^^,^^50^ For example, an intervention using points earned to capture usage, reported that over 70% of points were earned during the first week of the intervention, and system notifications prompting parents to watch application contents appeared to briefly boost the number of points earned.^48^ Predetermined criteria were occasionally used to define active and nonactive users.^29^^,^^51^ In 1 study, a participant was considered an active participant if they completed the presurvey and postsurvey and answered at least 1 program question, which 12 of 109 participants achieved.^29^ Fiks et al^51^ defined active engagement at the group level as at least 2 Facebook posts/comments per group per day, and all groups met these criteria.

Two studies related usage results to lifestyle outcomes.^51^^,^^70^ One study found that higher Facebook use (total comments and posts) was positively related to sleep duration, but not to other parameters.^70^ In another study, higher levels of individual usage were associated with lower weight-for-length *z*-scores in children.^51^ Furthermore, an association was found between lower levels of usage and parental characteristics (identifying as other than non-Hispanic White, completing less than a bachelor’s degree, and an annual household income < US$ 50,000).^42^

The results of self-reported usage (n=11) provided insights into participants’ self-estimated use of the intervention and reasons for (non)usage. For example, in 1 application intervention, participants reported seldom using the meal planning and grocery list features. Reasons included not knowing these features were available or lacking the desire to form new habits. Participants suggested making the shopping lists shareable among family members to increase usage.^41^ Other reasons for nonusage were technical problems, not understanding the intervention and a lack of interest in the intervention.^40^ For less educated participants, videos instead of written content could increase usage.^46^ Hull et al^40^ compared self-estimated usage with logged use data and concluded their results coincided.

### Other Outcomes

Other outcomes that were less frequently evaluated were usability (n=12), implementation (n=7), operability (n=2), adoption (n=1), cost-effectiveness (n=1), and dissemination (n=1). Usability was evaluated across all eHealth modalities, most commonly focusing on whether the modality was easy to use and understand for participants.^18^^,^^39^^,^^40^^,^^47^, ^48^, ^49^^,^^52^^,^^54^^,^^62^^,^^63^^,^^73^ Ease of use was further investigated by evaluating the user’s ability to find a specific part of the intervention (eg, a video presentation or discussion board thread).^47^ Other usability outcome measures included the need for technical assistance, participants’ confidence in using the intervention, and the readability of the intervention’s content.^54^^,^^58^ Usability questions were asked through questionnaires, interviews, and focus groups. Most studies used self-made questionnaires, but 1 study used adapted questions from the Mobile Application Rating Scale.^18^ Intervention operability was evaluated by reporting technical problems through questionnaires and interviews.^60^^,^^63^

Implementation was measured across all modalities, but mostly in applications (n=4). Outcome measures focused on implementation as conceived, number of intended sessions delivered, and intervention delivered as planned.^18^^,^^33^^,^^40^^,^^47^^,^^51^^,^^60^^,^^65^ These outcome measures were all related to the implementation of the intervention in the research setting, but not its implementation in the real-world setting.

### Reported Strengths and Limitations of Evaluation Methods

The most reported strength of evaluation methods was the use of validated questionnaires and devices (eg, an accelerometer).^28^^,^^31^^,^^39^^,^^43^^,^^46^^,^^53^^,^^60^^,^^62^^,^^68^^,^^69^^,^^71^^,^^73^ Qualitative research was considered another strength because it provides the researcher with a rich and contextualized understanding of a topic,^56^ especially when combined with quantitative outcome measures.^33^ This also applies to complementing self-reported usage data with logged usage data.^40^^,^^49^

As expected, authors reported unvalidated questionnaires as an important limitation.^54^^,^^62^^,^^67^ Another limitation of the use of questionnaires is the risk of bias (eg, measurement, social desirability, recall, and response bias).^27^^,^^30^^,^^31^^,^^36^^,^^40^^,^^45^^,^^46^^,^^50^^,^^60^^,^^62^^,^^64^, ^65^, ^66^^,^^69^^,^^71^ A lack of objective application usage data and lifestyle parameters (eg, physical activity measured by an actigraphy) was reported as a limitation for usage and effectiveness outcomes, respectively.^31^^,^^43^^,^^44^^,^^55^^,^^59^^,^^64^ However, collecting objective measurements was sometimes complicated by the digital nature of the intervention, necessitating the use of self-reported data.^49^

Some studies provided advice to improve evaluation methods. For example, to reduce social desirability bias, 1 study had a separate research organization conduct the interviews.^33^^,^^52^ The need for long-term outcomes to fully assess the potential of eHealth interventions was occasionally emphasized.^31^^,^^52^^,^^59^ Additionally, follow-up periods should match with the outcome measures; for example, 6 months was considered too brief to observe significant BMI changes in children.^66^^,^^69^ In addition, evaluation questionnaires should have a short completion time and be easy to administer to make study assessments feasible.^18^^,^^31^^,^^62^

## Discussion

The aim of this scoping review was to provide recommendations for the evaluation of eHealth lifestyle interventions, specifically targeting preschool children. Through the implementation of these recommendations, we aim to improve future evaluation processes, thereby advancing the development of improved eHealth lifestyle interventions.

### Principal Findings

This review included 48 articles, describing different development stages, ranging from preprototype to scale-up, of 31 eHealth interventions targeting the lifestyle of preschool children. These interventions predominantly targeted diet and were mostly conveyed through applications and websites. Most interventions were designed for use by parents to facilitate lifestyle changes in their children; however, 2 interventions were intended for direct use by preschool children themselves.

Of the RCTs that evaluated effectiveness in our review, 2 were in the demonstration phase, and the others were in the pilot phase. No RCT targeting children’s physical activity found significant improvements, whereas RCTs targeting diet found promising outcomes. However, studies with long-term results were limited, of which only 2 interventions reported moderate effects on sleep and diet. Although short-term results may appear positive, achieving long-term success could be more challenging owing to the time required to form lasting habits. For example, Lally et al^74^ found that forming a habit (eating, drinking, or physical exercise carried out daily in the same context) took participants 18 to 254 days. Moreover, the transtheoretical model of health behavior change suggests that maintenance of health behavior change will start only after 6 months.^75^

Unfortunately, no consensus was reached on the optimal duration for an acceptable and effective eHealth lifestyle intervention aimed at preschool children. In 5 studies, participants found their intervention’s duration (1-3 months) acceptable, whereas, in a 12-month study, nearly half of the participants preferred a shorter duration. Intervention acceptability was often centered on users’ affective attitudes, such as the intervention’s interestingness, a frequently used definition of acceptability in literature.^17^

Furthermore, studies reported that intervention usage naturally declined over time, a phenomenon commonly observed in eHealth trials.^76^ Additionally, results reported varying levels of usage across participants and intervention features. Therefore, some studies attempted to correlate usage data (eg, total usage and specific feature use) with lifestyle outcomes or participants’ characteristics. These findings could guide further eHealth development.

### Recommendations

**Figure 3:**
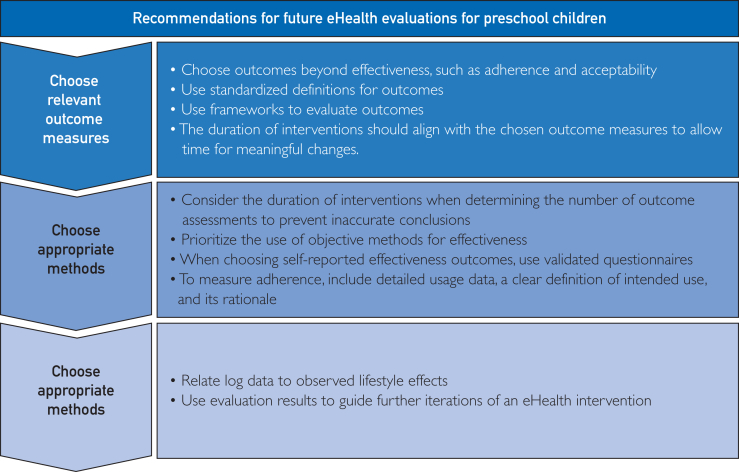
Recommendations for the evaluation of eHealth lifestyle interventions targeting preschool children.

On the basis of our findings, we present 10 recommendations for future evaluation of eHealth interventions. Our recommendations relate to 3 domains: the selection of relevant outcome measures and appropriate evaluation methods, and the integration and application of the evaluation results (Figure 3).

First, many of the included studies evaluated the effectiveness of their intervention. Although this is a crucial aspect of the digital health lifecycle, earlier stages assessing usability, acceptability, and operability provide valuable insights for improving the design, implementation, adoption, and utilization of eHealth interventions and should not be overlooked.^15^^,^^17^^,^^25^ Outcomes such as cost-effectiveness and adoption were evaluated only once, although these, alongside adherence and dissemination, reflect real-world implementation of interventions and are therefore important to evaluate.^77^ The limited evaluation of outcomes beyond effectiveness aligns with findings from the scoping review of reviews evaluating e- and mHealth lifestyle interventions for children by Kracht et al.^19^^,^^37^^,^^77^ Consistent with their suggestion, our first recommendation is that researchers of eHealth interventions targeting preschool children evaluate outcomes beyond effectiveness. Using frameworks that encourage researchers to assess outcomes beyond effectiveness, like the RE-AIM (Reach, Effectiveness, Adoption, Implementation and Maintenance) framework, could be beneficial.^37^

Second, the lack of consensus on the definitions of the evaluated outcomes, such as acceptability,^17^^,^^78^ became evident when comparing findings across the reviewed articles. For example, Kitsaras et al^39^ assessed acceptability through affective attitude, whereas Hammersley et al^69^ additionally assessed acceptability through perceived intervention effectiveness and perceived amount of effort required to participate in the intervention. Thus, our second recommendation is for researchers to adopt standardized definitions for their outcomes if available, to ensure consistency, comparability, and reliability of results across different studies. In Supplemental Appendix 3, definitions for the different outcome measures used in this scoping review are shown, supported by recent literature.

Third, aligning with the theoretical framework of acceptability,^78^ we observed that intervention acceptability was predominantly evaluated through users’ experienced affective attitudes. However, it can also be measured before participation (anticipated affective attitudes), to identify which aspects of the intervention could be adjusted to enhance acceptability and, consequently, participation.^78^ The focus on evaluating users’ experienced affective attitudes toward digital health interventions, rather than anticipated affective attitudes, has also been observed in another review.^79^ Considering that users’ experienced affective attitudes toward the interventions were generally positive, including other outcome measures might offer a clearer evaluation of acceptability. For example, attrition and retention rates, along with reasons for dropout, provided insights into user behavior, which can also indicate intervention acceptability. However, sole reliance on observed behavior does not explain, which aspects of the intervention were not acceptable. Moreover, we found that some dropout reasons, such as illness, were unrelated to intervention acceptability. In addition, sometimes, the results of the acceptability outcome measures disagreed with each other. For example, Helle et al^46^ reported that although most of their users found the intervention well adapted to their child’s age, easy to understand, and relevance, their attrition rate was higher than expected. Taken together, these points highlight the complexity of evaluating acceptability and underscore the need for a framework with multiple dimensions of an outcome. Therefore, as our third recommendation, we advise using outcome-specific frameworks for the evaluation of eHealth lifestyle interventions targeting preschool children because they can contribute to a more comprehensive evaluation.

In addition, 2 important considerations should be made in conjunction with the study duration: the selection of outcome measures and the timing of their evaluation. Among the 6 interventions that assessed BMI, only 1 found a significant improvement among preschool children after 6 months, whereas 2 interventions suggested that a 6-month period might be insufficient for an intervention to significantly improve preschool children’s BMI. Second, as anticipated, several studies reported a decline in intervention usage over time, whereas studies relying on a single assessment during a lengthy intervention may fail to capture this. Therefore, as our fourth recommendation, we advise that the duration of lifestyle interventions for preschool children be carefully considered in conjunction with the selected effectiveness outcome measures, to ensure sufficient time for meaningful changes to be detected. Moreover, as our fifth recommendation, we advise to account for the duration of lifestyle interventions for preschool children when deciding on the number of conducted outcome assessments, to avoid drawing inaccurate conclusions.

Furthermore, our findings indicate that the evaluation of effectiveness of eHealth lifestyle interventions targeting preschool children was predominantly based on parental-reported questionnaires. However, self-reported data are prone to response bias, as shown by Mazor et al.^80^ For example, participants in the intervention group may respond in a matter that aligns with their perception of the intervention’s intended outcome. Moreover, because parents often completed the questionnaires, responses may be less accurate because they might not fully account for the influence of others on their child’s lifestyle. For example, Young et al^81^ found that grandparents can negatively impact the child’s dietary intake. Consequently, our sixth recommendation is to prioritize the use of objective methods for effectiveness, such as pictures taken of plates with food for dietary outcomes, or an accelerometer for physical activity outcomes. Furhter, as our seventh recommendation, we advise the use of validated questionnaires (eg, the FFQ and EY-PAQ) when choosing self-reported effectiveness outcomes.

Additionally, evaluating adherence is important because it is needed to interpret effectiveness findings of eHealth lifestyle interventions. This requires a clear distinction between mere usage and adherence. Proper measurement of adherence requires (1) the usage data of participants; (2) a definition of the intended use; and (3) a rationale for the definition of the intended use.^82^ Intended use is an important element of adherence because it defines the usage threshold required to achieve the intervention’s aim (eg, form new habits).^82^ None of the studies included met all requirements to measure adherence. In 2 studies, active usage was defined without providing a rationale, and it was unclear whether authors meant adherence in this context. This finding aligns with other studies that found adherence is measured in various ways, often inconsistently across studies.^83^, ^84^, ^85^ This leads to our eighth recommendation: future evaluators of eHealth lifestyle interventions for preschool children should incorporate detailed usage data, provide a clear definition of intended use, and offer a rationale for this definition, to improve the measurement of adherence.

Following this, we found that incorporating detailed usage data can significantly enhance the understanding of how usage influences outcomes. For example, Fiks et al^51^ correlated individual participation, defined as the number of Facebook posts and comments, with the child’s weight-for-length *z*-score. The identified correlation can serve as a rationale for intended use in future studies.^82^ As reported by Ezran et al,^42^ log data can also be linked to participants’ characteristics, allowing for the identification of groups whose usage can be improved. Therefore, our ninth recommendation is to correlate log data with observed lifestyle effects to define the optimal usage necessary to reach the desired outcomes^86^ and to identify the most valuable intervention features.

Finally, yielded evaluation results offer insights into eHealth intervention features that did not meet the user’s needs, were ineffective and were not engaging for the target audience. Adjusting those features in further iterations could lead to higher acceptability or usability, potentially increasing the interventions’ effectiveness. Therefore, it is important to collect and review data regularly throughout the digital health intervention’s lifecycle.^25^ For example, evaluation during the prototype phase of the Hospital Hero application revealed issues with certain features, including excessive text and the absence of rewards after task completion. These findings informed the design of an improved iteration of the application for the pilot phase. Accordingly, our tenth recommendation is to use evaluation results to guide further iterations of eHealth lifestyle interventions for preschool children.

### Limitations

Although we provided an elaborate overview of evaluated outcome measures and corresponding evaluation methods of eHealth lifestyle interventions for preschool children, certain limitations should be acknowledged. First, the heterogeneity of the included interventions (eg, modalities, targeted lifestyle component, and duration) might limit the generalizability of our findings across different interventions. To minimize this, we structured our outcomes according to the modality or lifestyle component, depending on its relevance to the evaluated outcome. Second, in many studies a single construct of acceptability was evaluated (eg, user’s experienced affective attitude). However, this narrow focus does not unequivocally signify intervention acceptability. Therefore, the acceptability results presented in this review may not fully capture the comprehensive nature of how interventions are perceived by users. Furthermore, some outcomes were rarely evaluated (eg, cost-effectiveness and dissemination), which prevented us from providing detailed insights into these areas. This absence likely arises because these outcome measures are more prevalent in the implementation phase of eHealth interventions, whereas most included were still in the design or evaluation phase.

## Conclusion

This scoping review examined how eHealth lifestyle interventions for preschool children are being evaluated, highlighting the strengths and limitations of the evaluation methods, as well as the results of these assessments. Although short-term effectiveness, particularly in diet-related behaviors, appear promising, there remains limited evidence on the long-term effectiveness of these interventions, particularly in areas like physical activity and sleep.

Our findings resulted in 10 recommendations for the evaluation of eHealth interventions for preschool children, aiming to contribute to the development of improved eHealth lifestyle interventions for preschool children. These recommendations relate to 3 domains: the selection of relevant outcome measures and appropriate evaluation methods, and the integration and application of the evaluation results. Specifically, this review stressed the importance of evaluating outcomes beyond effectiveness because this could help advance our understanding of eHealth interventions and their successes in improving the lifestyle of preschool children.

## Potential Competing Interests

Dr Andrea Evers reports research grants (paid to institution) from the European Research Council, Dutch Research Organisation (NWO), and Dutch health charity associations (eg, Hartstichting, Diabetesfonds, ReumaNL, and Nierstichting) as well as private partners. This publication is part of the project Features of Lifestyle in Young Kids (FLY-Kids) funded by the Dutch ministry of Health, Welfare and Sports (in Dutch: Ministerie van Volksgezondheid, Welvaart en Sport [VWS]). The other authors report no competing interests.

## Declaration of Generative AI and AI-Assisted Technologies in the Writing Process

During the preparation of this work, the authors used ChatGPT to improve language and readability. After using this tool/service, the authors reviewed and edited the content as needed and takes full responsibility for the content of the publication.
