# Correction Notices

**DOI:** 10.1016/j.mcpdig.2025.100305

**Published:** 2025-11-07

Corrigendum to “Exploring Evaluation of eHealth Lifestyle Interventions for Preschool Children: A Scoping Review” *Mayo Clinic Proceedings: Digital Health Volume 3 (2025), 100223*

Marissa C.J. Kooij, MD; Ashley J.P. Smit, MSc; Linda D. Breeman, PhD; Lieke Schiphof-Godart, PhD; Isra Al-Dhahir, MSc; Andrea W.M. Evers, PhD; and Koen F.M. Joosten, MD, PhD

Department of Neonatal and Pediatric Intensive Care (M.C.J.K., A.J.P.S., K.F.M.J.) and Department of Medical Informatics (L.S-G.), Erasmus MC Sophia Children’s Hospital, Rotterdam, Netherlands; and Health, Medical, and Neuropsychology Unit (L.D.B., I.A-D., A.W.M.E.,), Leiden University, Netherlands.

The authors regret that the unfinished version of Figure 3 was published as an error. The final version has now been posted online. The authors would like to apologize for any inconvenience caused.

DOI of original article: 10.1016/j.mcpdig.2025.100223

**Correspondence:** Address to Marissa C.J. Kooij, MD, Department of Neonatal and Pediatric Intensive Care, Erasmus MC Sophia Children’s Hospital, Rotterdam, Netherlands. (m.kooij@erasmusmc.nl).

