# Supplementary material for: Exploring Evaluation of eHealth Lifestyle Interventions for Preschool Children: A Scoping Review
Source: Mayo Clin Proc Digit Health. 2025 Apr 17;3(2):100223. doi: 10.1016/j.mcpdig.2025.100223 (PMC12190898; doi:10.1016/j.mcpdig.2025.100223)
Supplement: Supplementary Data [file mmc1.pdf]

Supplemental appendices

Exploring Evaluation of eHealth Lifestyle Interventions for Preschool Children: A Scoping Review

Table of contents

Supplemental appendix 1. Search strategy **3**
Supplemental appendix 2. Intervention characteristics **6**
Supplemental appendix 3. Definitions of outcomes in this scoping review **16**
Supplemental table 1. Outcome effectiveness **17**
Supplemental table 2. Outcome acceptability **19**
Supplemental table 3. Outcome usage **20**
References **21**

Supplemental appendix 1. Search strategy

Table 1. Overview of search strategy				
**Database searched**	**Platform**	**Years of coverage**	**Records**	**Records after duplicates removed**
Medline ALL	Ovid	1946 - 2023	1897	1891
Embase	Embase.com	1971 - 2023	2846	1510
Web of Science Core Collection*	Web of Knowledge	1975 - 2023	1671	475
CINAHL**	EBSCO	1982 - 2023	811	212
Additional Search Engines: Google Scholar***			200	168
**Total**			**7425**	**4256**

*Science Citation Index Expanded (1975-present) ; Social Sciences Citation Index (1975-present) ; Arts & Humanities Citation Index (1975-present) ; Conference Proceedings Citation Index- Science (1990-present) ; Conference Proceedings Citation Index- Social Science & Humanities (1990-present) ; Emerging Sources Citation Index (2005-present)
**Limited to Academic Journals
***Google Scholar was searched via "Publish or Perish" to download the results in EndNote.

No other database limits were used than those specified in the search strategies

medline

(Life Style/ OR exp Smoking/ OR exp Diet/ OR Eating/ OR exp Exercise/ OR exp Alcohol Drinking/ OR Stress, Psychological/ OR exp blood Pressure/ OR Body Weight/ OR exp Body Weight Changes/ OR Ideal Body Weight/ OR Body Mass Index/ OR exp Sleep/ OR exp Smoking Cessation/ OR Blood Pressure Determination/ OR Maternal Behavior/ OR Paternal Behavior/ OR (lifestyle* OR life-style* OR smoking* OR tobacco* OR diet* OR exercise* OR alcohol* OR stress* OR (blood ADJ3 pressure*) OR (body ADJ3 (weight OR mass)) OR (weight ADJ3 (change* OR gain* OR reduc* OR loss* OR loosing)) OR bmi OR sleep* OR overweight* OR obesit* OR (physical* ADJ3 (activ* OR inactiv*)) OR sedentary OR hypertens* OR ((parent* OR paternal* OR maternal* OR father* OR mother*) ADJ3 (behav* OR healthy))).ab,ti,kw.) **AND** (Online Systems / OR Telemedicine / OR Mobile Applications / OR Cell Phone / OR Internet-Based Intervention / OR Text Messaging / OR Social Media / OR online system/ OR (telehealth* OR ehealth* OR tele-health* OR e-health* OR mobile-health* OR (digital ADJ3 (health* OR intervention*)) OR m-health* OR mhealth* OR (mobile ADJ3 app*) OR smartphone* OR smart-phone* OR mobile-phone* OR cell*-phone* OR cellphone* OR web-base* OR webbase* OR internet-base* OR text-messag* OR sms OR social-media OR twitter OR instagram OR facebook OR tiktok OR whatsapp* OR telemedicine* OR telephone-base*).ab,ti,kw. OR (app OR apps).ti.) **AND** (*"Child"/ OR "Infant"/ OR "Child, Preschool"/ OR (young-famil* OR ((4 OR four OR 1 OR one OR 2 OR two OR 3 OR three) ADJ4 (year OR years) ADJ3 (child* OR toddler* OR infant* OR baby OR babies OR preschool*)) OR ((1 OR 2 OR 3 OR 4 OR 5 OR 6 OR 7 OR 8 OR 9 OR 10 OR 11 OR 12 OR 13 OR 14 OR 15 OR 16 OR 17 OR 18 OR 19 OR 20 OR 21 OR 22 OR 23 OR 24 OR one OR two OR three OR four OR five OR six OR seven OR eight OR nine OR ten OR eleven OR twelve OR thirteen OR fourteen OR fifteen OR sixteen OR seventeen OR eighteen OR nineteen OR twenty OR twentyone OR twentytwo OR twentythree OR twentyfour) ADJ4 (month*) ADJ3 (child* OR toddler* OR infant* OR baby OR babies OR preschool* OR newborn* OR new-born*)) OR ((family OR parent* OR mother* OR father*) ADJ6 (pediatric* OR paedatric* OR toddler* OR infant* OR baby OR babies OR preschool* OR pre-school* OR young-child* OR newborn* OR new-born*)) OR ((child*) ADJ3 (younger OR under OR age OR aged OR old) ADJ2 (1 OR 2 OR 3 OR 4 OR 5 OR 6 OR 7 OR 8 OR 9 OR 10 OR 11 OR 12 OR one OR two OR three OR four OR five OR six OR seven OR eight OR nine OR ten OR eleven OR twelve) ADJ2 (year*))).ab,ti,kf.) **AND** english.la. **NOT** (exp animals/ NOT humans/)

embase

(lifestyle/exp OR 'lifestyle modification'/de OR smoking/exp OR diet/exp OR 'dietary intake'/exp OR exercise/exp OR 'alcohol consumption'/de OR 'mental stress'/exp OR 'blood pressure'/exp OR 'body weight'/de OR 'body weight change'/exp OR 'body weight control'/exp OR 'ideal body weight'/exp OR 'lean body weight'/exp OR 'liveweight gain'/exp OR 'body mass'/de OR sleep/exp OR 'smoking cessation'/de OR 'blood pressure monitoring'/de OR 'physical activity'/de OR 'parental behavior'/de OR 'maternal behavior'/de OR 'paternal behavior'/de OR (lifestyle* OR life-style* OR smoking* OR tobacco* OR diet* OR exercise* OR alcohol* OR stress* OR (blood NEAR/3 pressure*) OR (body NEAR/3 (weight OR mass)) OR (weight NEAR/3 (change* OR gain* OR reduc* OR loss*

OR loosing)) OR bmi OR sleep* OR overweight* OR obesit* OR (physical* NEAR/3 (activ* OR inactiv*)) OR sedentary OR hypertens* OR ((parent* OR paternal* OR maternal* OR father* OR mother*) NEAR/3 (behav* OR healthy))):ab,ti,kw) **AND** (telehealth/de OR telemedicine/de OR 'mobile application'/exp OR 'mobile phone'/exp OR 'web-based intervention'/de OR 'text messaging'/de OR 'social media'/de OR 'online system'/de OR 'mhealth'/exp OR 'mobile health'/exp OR 'mobile health application'/exp OR 'telemonitoring'/exp OR 'digital health'/exp OR 'digital health technology'/exp OR 'digital health intervention'/exp OR (telehealth* OR ehealth* OR tele-health* OR e-health* OR mobile-health* OR (digital NEAR/3 (health* OR intervention*)) OR m-health* OR mhealth* OR (mobile NEAR/3 app*) OR smartphone* OR smart-phone* OR mobile-phone* OR cell*-phone* OR cellphone* OR web-base* OR webbase* OR internet-base* OR text-messag* OR sms OR social-media OR twitter OR instagram OR facebook OR tiktok OR whatsapp* OR telemedicine* OR telephone-base*):Ab,ti,kw OR (app OR apps):ti) **AND** ('child'/de/mj OR 'infant'/exp OR 'toddler'/exp OR 'preschool child'/exp OR (young-famil* OR ((4 OR four OR 1 OR one OR 2 OR two OR 3 OR three) NEAR/4 (year OR years) NEAR/3 (child* OR toddler* OR infant* OR baby OR babies OR preschool*)) OR ((1 OR 2 OR 3 OR 4 OR 5 OR 6 OR 7 OR 8 OR 9 OR 10 OR 11 OR 12 OR 13 OR 14 OR 15 OR 16 OR 17 OR 18 OR 19 OR 20 OR 21 OR 22 OR 23 OR 24 OR one OR two OR three OR four OR five OR six OR seven OR eight OR nine OR ten OR eleven OR twelve OR thirteen OR fourteen OR fifteen OR sixteen OR seventeen OR eighteen OR nineteen OR twenty OR twentyone OR twentytwo OR twentythree OR twentyfour) NEAR/4 (month*) NEAR/3 (child* OR toddler* OR infant* OR baby OR babies OR preschool* OR newborn* OR new-born*)) OR ((family OR parent* OR mother* OR father*) NEAR/6 (pediatric* OR paedatric* OR toddler* OR infant* OR baby OR babies OR preschool* OR pre-school* OR young-child* OR newborn* OR new-born*)) OR ((child*) NEAR/3 (younger OR under OR age OR aged OR old) NEAR/2 (1 OR 2 OR 3 OR 4 OR 5 OR 6 OR 7 OR 8 OR 9 OR 10 OR 11 OR 12 OR one OR two OR three OR four OR five OR six OR seven OR eight OR nine OR ten OR eleven OR twelve) NEAR/2 (year*))):ab,ti,kw) NOT ([conference abstract]/lim AND [2000-2019]/py) AND [english]/lim NOT ([animals]/lim NOT [humans]/lim)

Web of science

TS=(((lifestyle* OR life-style* OR smoking* OR tobacco* OR diet* OR exercise* OR alcohol* OR stress* OR (blood NEAR/2 pressure*) OR (body NEAR/2 (weight OR mass)) OR (weight NEAR/2 (change* OR gain* OR reduc* OR loss* OR loosing)) OR bmi OR sleep* OR overweight* OR obesit* OR (physical* NEAR/2 (activ* OR inactiv*)) OR sedentary OR hypertens* OR ((parent* OR paternal* OR maternal* OR father* OR mother*) NEAR/2 (behav* OR healthy)))) **AND** ((telehealth* OR ehealth* OR tele-health* OR e-health* OR mobile-health* OR (digital NEAR/2 (health* OR intervention*)) OR m-health* OR mhealth* OR (mobile NEAR/2 app*) OR smartphone* OR smart-phone* OR mobile-phone* OR cell*-phone* OR cellphone* OR web-base* OR webbase* OR internet-base* OR text-messag* OR sms OR social-media OR twitter OR instagram OR facebook OR tiktok OR whatsapp* OR telemedicine* OR telephone-base*)) **AND** ((young-famil* OR ((4 OR four OR 1 OR one OR 2 OR two OR 3 OR three) NEAR/4 (year OR years) NEAR/3 (child* OR toddler* OR infant* OR baby OR babies OR preschool*)) OR ((1 OR 2 OR 3 OR 4 OR 5 OR 6 OR 7 OR 8 OR 9 OR 10 OR 11 OR 12 OR 13 OR 14 OR 15 OR 16 OR 17 OR 18 OR 19 OR 20 OR 21 OR 22 OR 23 OR 24 OR one OR two OR three OR four OR five OR six OR seven OR eight OR nine OR ten OR eleven OR twelve OR thirteen OR fourteen OR fifteen OR sixteen OR seventeen OR eighteen OR nineteen OR twenty OR twentyone OR twentytwo OR twentythree OR twentyfour) NEAR/4 (month*) NEAR/3 (child* OR toddler* OR infant* OR baby OR babies OR preschool* OR newborn* OR new-born*)) OR ((family OR parent* OR mother* OR father*) NEAR/6 (pediatric* OR paedatric* OR toddler* OR infant* OR baby OR babies OR preschool* OR pre-school* OR young-child* OR newborn* OR new-born*)) OR ((child*) NEAR/3 (younger OR under OR age OR aged OR old) NEAR/2 (1 OR 2 OR 3 OR 4 OR 5 OR 6 OR 7 OR 8 OR 9 OR 10 OR 11 OR 12 OR one OR two OR three OR four OR five OR six OR seven OR eight OR nine OR ten OR eleven OR twelve) NEAR/2 (year*))))) **NOT** DT=(Meeting Abstract OR Meeting Summary) **AND** LA=(English)

Cinahl

(MH Life Style OR MH Life Style, Sedentary OR MH Life Style Changes OR MH Smoking+ OR MH Diet+ OR MH Eating OR MH Exercise+ OR MH Alcohol Drinking+ OR MH Stress, Psychological+ OR MH blood Pressure+ OR MH Body Weight OR MH Body Weight Changes+ OR MH Body Mass Index OR MH Sleep+ OR MH Smoking Cessation+ OR MH Blood Pressure Determination OR MH Maternal Behavior OR MH Paternal Behavior

OR TI(lifestyle* OR life-style* OR smoking* OR tobacco* OR diet* OR exercise* OR alcohol* OR stress* OR (blood N2 pressure*) OR (body N2 (weight OR mass)) OR (weight N2 (change* OR gain* OR reduc* OR loss* OR loosing)) OR bmi OR sleep* OR overweight* OR obesit* OR (physical* N2 (activ* OR inactiv*)) OR sedentary OR hypertens* OR ((parent* OR paternal* OR maternal* OR father* OR mother*) N2 (behav* OR healthy))) OR AB(lifestyle* OR life-style* OR smoking* OR tobacco* OR diet* OR exercise* OR alcohol* OR stress* OR (blood N2 pressure*) OR (body N2 (weight OR mass)) OR (weight N2 (change* OR gain* OR reduc* OR loss* OR loosing)) OR bmi OR sleep* OR overweight* OR obesit* OR (physical* N2 (activ* OR inactiv*)) OR sedentary OR hypertens* OR ((parent* OR paternal* OR maternal* OR father* OR mother*) N2 (behav* OR healthy)))) **AND** (MH Online Systems OR MH Telemedicine OR MH Mobile Applications OR MH Cellular Phone OR MH Internet-Based Intervention OR MH Text Messaging OR MH Social Media OR TI(telehealth* OR ehealth* OR tele-health* OR e-health* OR mobile-health* OR (digital N2 (health* OR intervention*)) OR m-health* OR mhealth* OR (mobile N2 app*) OR smartphone* OR smart-phone* OR mobile-phone* OR cell*-phone* OR cellphone* OR web-base* OR webbase* OR internet-base* OR text-messag* OR sms OR social-media OR twitter OR instagram OR facebook OR tiktok OR whatsapp* OR telemedicine* OR telephone-base*) OR AB(telehealth* OR ehealth* OR tele-health* OR e-health* OR mobile-health* OR (digital N2 (health* OR intervention*)) OR m-health* OR mhealth* OR (mobile N2 app*) OR smartphone* OR smart-phone* OR mobile-phone* OR cell*-phone* OR cellphone* OR web-base* OR webbase* OR internet-base* OR text-messag* OR sms OR social-media OR twitter OR instagram OR facebook OR tiktok OR whatsapp* OR telemedicine* OR telephone-base*) OR TI(app OR apps)) **AND** (MM "Child" MH "Child, Preschool" OR MH "Infant+" OR TI(young-famil* OR ((4 OR four OR 1 OR one OR 2 OR two OR 3 OR three) N4 (year OR years) N3 (child* OR toddler* OR infant* OR baby OR babies OR preschool*)) OR ((1 OR 2 OR 3 OR 4 OR 5 OR 6 OR 7 OR 8 OR 9 OR 10 OR 11 OR 12 OR 13 OR 14 OR 15 OR 16 OR 17 OR 18 OR 19 OR 20 OR 21 OR 22 OR 23 OR 24 OR one OR two OR three OR four OR five OR six OR seven OR eight OR nine OR ten OR eleven OR twelve OR thirteen OR fourteen OR fifteen OR sixteen OR seventeen OR eighteen OR nineteen OR twenty OR twentyone OR twentytwo OR twentythree OR twentyfour) N4 (month*) N3 (child* OR toddler* OR infant* OR baby OR babies OR preschool* OR newborn* OR new-born*)) OR ((family OR parent* OR mother* OR father*) N6 (pediatric* OR paedatric* OR toddler* OR infant* OR baby OR babies OR preschool* OR pre-school* OR young-child* OR newborn* OR new-born*)) OR ((child*) N3 (younger OR under OR age OR aged OR old) N2 (1 OR 2 OR 3 OR 4 OR 5 OR 6 OR 7 OR 8 OR 9 OR 10 OR 11 OR 12 OR one OR two OR three OR four OR five OR six OR seven OR eight OR nine OR ten OR eleven OR twelve) N2 (year*))) OR AB(young-famil* OR ((4 OR four OR 1 OR one OR 2 OR two OR 3 OR three) N4 (year OR years) N3 (child* OR toddler* OR infant* OR baby OR babies OR preschool*)) OR ((1 OR 2 OR 3 OR 4 OR 5 OR 6 OR 7 OR 8 OR 9 OR 10 OR 11 OR 12 OR 13 OR 14 OR 15 OR 16 OR 17 OR 18 OR 19 OR 20 OR 21 OR 22 OR 23 OR 24 OR one OR two OR three OR four OR five OR six OR seven OR eight OR nine OR ten OR eleven OR twelve OR thirteen OR fourteen OR fifteen OR sixteen OR seventeen OR eighteen OR nineteen OR twenty OR twentyone OR twentytwo OR twentythree OR twentyfour) N4 (month*) N3 (child* OR toddler* OR infant* OR baby OR babies OR preschool* OR newborn* OR new-born*)) OR ((family OR parent* OR mother* OR father*) N6 (pediatric* OR paedatric* OR toddler* OR infant* OR baby OR babies OR preschool* OR pre-school* OR young-child* OR newborn* OR new-born*)) OR ((child*) N3 (younger OR under OR age OR aged OR old) N2 (1 OR 2 OR 3 OR 4 OR 5 OR 6 OR 7 OR 8 OR 9 OR 10 OR 11 OR 12 OR one OR two OR three OR four OR five OR six OR seven OR eight OR nine OR ten OR eleven OR twelve) N2 (year*)))) AND LA(english) NOT (MH animals+ NOT MH humans+)

Source type: Academic journals

Google scholar

lifestyle|"life style telehealth|ehealth|"e|mobile health"|"mobile app|application"|smartphone infant|infants|preschool|baby|babies|"young child|children"

lifestyle|'life style' telehealth|ehealth|'e|mobile health'|'mobile app|application'|smartphone infant|infants|preschool|baby|babies|'young child|children'

Supplemental appendix 2. Intervention characteristics

Table 1. Intervention characteristics

Study name	Country	First author, year of publication	Study design)	Intervention used by children, parents, or both	Number of participants	Age child	Targeted lifestyle component(s)	Modality	Description of the intervention	Duration of intervention	Controlgroup description
Babysleep website[1]	USA	Mindell J, 2021	Observational	Parents	842,87	0-36 months	Sleep	Website	The website provides pediatric sleep education in both written and video formats. It ensures privacy by not requiring account logins or collecting user data. The site is divided into three sections: educational materials on sleep by age and category, a directory of resources and sleep centers, and updates on sleep-related research and news.	N.A.	N.A.
Bedtime Routines Intervention for Children (BRIC)[2]	UK	Kitsaras G, 2022	Quasi-experimental	Parents	50	1-3 years	Sleep	SMS	The intervention consisted of a series of text messages sent to parents for seven consecutive nights, arriving about one hour before their child's usual bedtime. Each message was unique and included content based on behavior change techniques (BCTs) to promote engagement in optimal bedtime routines. Participants had the option to choose between a detailed version or a shorter, summary version of each message.	7 days	N.A.
Children Eating Well[3]	USA	Hull P, 2017	Quasi-experimental	Parents	63	2-4 years	Nutrition	App	The app includes WIC shopping tools to simplify the shopping process and nutrition education for parents of 2- to 4-year-olds, with content available in both English and Spanish. Push notifications deliver messages, including features like a Yummy Snack Gallery and Healthy Snacking Tips, on a predetermined schedule, with each message sent twice over the 3-month testing period.	3 months	N.A.
Cooking Matters Facebook Platform[4]	USA	Zhang Q, 2021	Quasi-experimental	Parents	397	0-5 years	Nutrition	Facebook	The Facebook page regularly publishes seasonal recipes, tips for food planning and cooking, related videos, and live events. Social posts focus on easy, healthy recipes to prepare on a limited budget, live events that include recipe and skill demos from experienced instructors, and a variety of videos addressing caregivers’ barriers to healthy eating and encouraging positive behaviors like meal planning, involving kids in the kitchen, and making healthy foods available for snacking.	2 months	N.A.

Cooking Matters Mobile Application[5]	USA	Garvin TM, 2019	Quasi-experimental	Parents	461 (survey) and 20 (interview)	pregnancy /infant - 5 years	Nutrition	App	The app helped with meal planning and preparation, with features based on skills taught in the Cooking Matters course: recipes, shopping list and meal planning.	N.A. (Current users were asked for participation in surveys and interviews.)	N.A.
Creating Healthy Habits Among Maryland Preschoolers (CHAMP)[6]	USA	Ezran M, 2021	Quasi-experimental evaluation within RCT	Parents	261 children in 17 childcare centers, 17 parents	3-5	Nutrition, physical activity. And parenting/ welness topics	Website, e-mail or SMS	The website features a home and center-specific page with infographics and short videos offering nutritious recipes, strategies for introducing new foods, tips for managing picky eating, family-friendly physical activities, and guidance on sleep and stress management. It also includes challenges for parents, such as trying new foods or playing a sport with their child, with incentives for participation. Weekly e-mails or texts notify parents of new website content updates.	The intervention lasted 29 or 33 weeks.	The control arm received intervention materials the following year.
Early Food for Future Health[7]	Norway	Helle C, 2019	RCT	Parents	715	3-5 months	Nutrition	Website	Parents received an e-mail each month from child age 6 to 12 months with a link to the age-appropriate webpage showing the month’s video clip on the infant feeding topic together with the corresponding recipes and cooking-films.	7 months	Parents in the control group received routine care from their local child health clinic with regular consultations at child age 6, 8, 10 and 12 months.
Early Food for Future Health[8]	Norway	Helle C, 2019	RCT	Parents	715	3-5 months	Nutrition	Website	Parents received an e-mail each month from child age 6 to 12 months with a link to the age-appropriate webpage showing the month’s video clip on the infant feeding topic together with the corresponding recipes and cooking-films.	7 months	Parents in the control group received routine care from their local child health clinic with regular consultations at child age 6, 8, 10 and 12 months.
Enabling Mothers to Prevent Pediatric Obesity Through Web-Based Education and Reciprocal Determinism (EMPOWER)[9]	USA	Knowlden A, 2015	RCT	Parents	57	4-6 years	Nutrition, physical activity, screen time	Website	The EMPOWER intervention used a social cognitive theory framework. It included five educational sessions, each dedicated to one behavior in prevention of childhood obesity, using audiovisual presentations, interactive worksheets, and discussion boards, with a comprehensive booster session provided between posttest and follow-up.	4 weeks, with a booster session delivered within the 4 weeks after this.	The Healthy Lifestyles served as the active control intervention, designed to provide mothers with knowledge-based education on preventing childhood obesity. It addressed the same behaviors as in the intervention group, using similar modalities like short presentations, worksheets, and discussion board activities, with a follow-up booster session included.
Enabling Mothers to Prevent Pediatric Obesity Through Web-Based Education and Reciprocal Determinism (EMPOWER)[10]	USA	Knowlden A, 2014	RCT	Parents	57	4-6 years	Nutrition, physical activity, screen time	Website	The EMPOWER intervention used a social cognitive theory framework. It included five educational sessions, each dedicated to one behavior in prevention of childhood obesity, using audiovisual presentations, interactive worksheets, and discussion boards, with a comprehensive booster session provided between posttest and follow-up.	4 weeks, with a booster session delivered within the 4 weeks after this.	The Healthy Lifestyles served as the active control intervention, designed to provide mothers with knowledge-based education on preventing childhood obesity. It addressed the same behaviors as in the intervention group, using

											similar modalities like short presentations, worksheets, and discussion board activities, with a follow-up booster session included.
Enabling Mothers to Prevent Pediatric Obesity Through Web-Based Education and Reciprocal Determinism (EMPOWER)[11]	USA	Knowlden A, 2016	RCT (1-year follow-up)	Parents	57	4-6 years	Nutrition, physical activity, screen time	Website	The EMPOWER intervention used a social cognitive theory framework. It included five educational sessions, each dedicated to one behavior in prevention of childhood obesity, using audiovisual presentations, interactive worksheets, and discussion boards, with a comprehensive booster session provided between posttest and follow-up.	4 weeks, with a booster session delivered within the 4 weeks after this.	The Healthy Lifestyles served as the active control intervention, designed to provide mothers with knowledge-based education on preventing childhood obesity. It addressed the same behaviors as in the intervention group, using similar modalities like short presentations, worksheets, and discussion board activities, with a follow-up booster session included.
Enabling Mothers to Prevent Pediatric Obesity Through Web-Based Education and Reciprocal Determinism (EMPOWER)[12]	USA	Knowlden A, 2018	RCT (2-year follow-up)	Parents	57	4-6 years	Nutrition, physical activity, screen time	Website	The EMPOWER intervention used a social cognitive theory framework. It included five educational sessions, each dedicated to one behavior in prevention of childhood obesity, using audiovisual presentations, interactive worksheets, and discussion boards, with a comprehensive booster session provided between posttest and follow-up.	4 weeks, with a booster session delivered within the 4 weeks after this.	The Healthy Lifestyles served as the active control intervention, designed to provide mothers with knowledge-based education on preventing childhood obesity. It addressed the same behaviors as in the intervention group, using similar modalities like short presentations, worksheets, and discussion board activities, with a follow-up booster session included.
Fammeal[13]	Portugal	Afonso L, 2020	Quasi-experimental	Parents and children	13 (acceptance test) and 21 (pilot RCT).	3-6 years	Nutrition, physical activity and sleep	App	The app included the recommendation system, i.e., the questionnaire to be filled out during the registration process, the cutoffs to tailor recommendations, and educational videos that appeared automatically as “recommended” or as “other videos” (users could access all the videos) based on parents’ answers to the questionnaire.	4 weeks	Parents in the control group were treated as usual, namely, by receiving recommendations to improve the family lifestyle in the well-child care visit given by the medical doctor.
Fammeal[14]	Portugal	Afonso L, 2020	RCT	Parents	35 (study 1) and 15 (study 2)	3-6 years	Nutrition, physical activity and sleep	App	The app included the recommendation system, i.e., the questionnaire to be filled out during the registration process, the cutoffs to tailor recommendations, and educational videos that appeared automatically as “recommended” or as “other videos” (users could access all the videos) based on parents’ answers to the questionnaire.	4 weeks	Parents in the control group were treated as usual, namely, by receiving recommendations to improve the family lifestyle in the well-child care visit given by the medical doctor.

Food4toddlers[15]	Norway	Røed M, 2020	Quasi-experimental evaluation within RCT	Parents	404	12 months	Nutrition	Website	The intervention group had 6 months of access to the Food4toddlers website which comprised 4 main elements: (1) lessons on how to provide healthy food and create a healthy eating environment for the toddler, (2) recipes, (3) a discussion forum, and (4) basic information about food and beverages (called *Good to know*). Intervention and control group.	6 months	No access to the website.
Food4toddlers[16]	Norway	Røed M, 2021	RCT	Parents	298	12 months	Nutrition	Website	The intervention group had 6 months of access to the Food4toddlers website which comprised 4 main elements: (1) lessons on how to provide healthy food and create a healthy eating environment for the toddler, (2) recipes, (3) a discussion forum, and (4) basic information about food and beverages (called *Good to know*). Intervention and control group.	6 months	No access to the website.
Grow2Gether[17]	USA	Fiks AG, 2017	RCT	Parents	85	0-9 months	Nutrition, sleep, screen time	Facebook	The intervention involved a private Facebook peer group, focused on healthy parenting and infant growth, facilitated by a psychologist specializing in obesity prevention. Participants were divided into small peer groups based on their infants' due dates, and the group activities centered around a video-based curriculum covering topics like infant feeding, sleep, positive parenting, and maternal well-being. Weekly videos were posted for the first six months, followed by biweekly posts, and the mothers engaged by sharing experiences, discussing parenting topics, and receiving feedback from both peers and the facilitator	11 months (2 months prenatal)	Participants in both the intervention and control groups received text message reminders for recommended infant primary care visits. The control group received no additional intervention.
Grow2gether[18]	USA	Gruver R, 2016	Quasi-experimental	Parents	29 (focus groups) and 8 (pilot trial)	0-12 months	Nutrition, sleep and physical activity	Facebook	The intervention involved a private Facebook peer group, with optional in-person meetings. Participants accessed weekly educational videos featuring mothers and infants from the community, modeling healthy behaviors related to infant growth. Mothers engaged by sharing their own content, providing feedback to peers, and receiving input from the group moderator, with additional weekly modules including a video and a summary of key points, alongside shorter posts with fun facts and health tips linked to external resources.	8 weeks	N.A.
Head start[19]	USA	Lawton K, 2022	Quasi-experimental	Parents	30	3-5 years	Nutrition	Facebook	The Facebook intervention spanned 3-weeks. Intervention posts were shared 5 days a week (Monday through Friday) and 2 to 3 times per day. Intervention posts were adapted from Sesame Street's Food for Thought: Eating Well on a Budget curriculum that was designed to support and educate parents of children between the ages of 2–8 years who may have limited access to affordable and nutritious food	3 weeks	N.A.
Healthy Beginnings[20]	USA	Evans MG, 2022	Quasi-experimental	Parents	109	0-5 years (according to participant characteristics table,	Nutrition, physical activity	SMS	12-week SMS-based health education program implemented at an early Head Start early childhood development center. Two uni-directional texts per week and one bi-directional. The uni-directional texts were either informational or encouraging. The bi-directional text was a question used to assess Theory of Planned Behavior constructs (attitudes, subjective norms, and perceived behavioral control).	12 weeks	N.A.

						not described in text)					
HEalthy EnviROnments (HEROs) intervention[21]	USA	Reyes LI, 2023	Quasi-experimental	Children	33	3-5 years	Nutrition and physical activity	App	The 4 HEROs apps—Tasting Party Express, Jungle Gym 1, Jungle Gym 2, and Spin-n-Move (Figure)—aimed to build food- and movement-based vocabulary, encourage new foods, support the progression of movement skills, and offered opportunities for PA. Tasting Party Express invited children to offer animated characters foods via a drag-and-drop action to build familiarity with food-based vocabulary and encourage trying new foods. Jungle Gym 1 and Jungle Gym 2 consisted of outdoor-like activities encouraging children to perform movement skills. Spin-n-Move involved a spinning wheel in which children are invited to perform movement skills on which the spinner randomly lands.	6 weeks	N.A.
Healthy Families, Healthy Kids 2–5 (HFHK2–5)[22]	USA	Davies MA, 2014	Quasi-experimental	Parents	10 (usability study) and 15 (feasibility study)	2-5 years	Nutrition, physical activity, screen time, sleep	Website	The final version of HFHK2–5 included a welcome page and nine web pages related to family history, child temperament, four child lifestyle factors (i.e., eating habits, physical activity, sedentary activity, and sleep habits), and three environmental factors (i.e., family practices during mealtime, family function, and the built environment).	N.A.	N.A.
Johnson's Bedtime Baby Sleep App[23]	USA	Leichman ES, 2020	Quasi-experimental	Parents	404	Infants (age not specified, infants included were 6 to 12 months)	Sleep	App	The Customized Sleep Profile (CSP) is an algorithm-based tool that allows a caregiver to complete a sleep analysis. Based on responses from the sleep analysis, the app provides psychoeducation and individually tailored recommendations, targeting areas such as bedtime, bedtime routine, parent behavior in response to night wakings, sleep duration, sleep space, and overall sleep health. The CSP is part of the Johnson's Bedtime Baby Sleep App, which also includes sleep tracking and an Ask the Expert function.	N.A. (Although there needed to be a minimum of 4 days and a maximum of 28 days between the first use (initial assessment) and second use of the CSP)	N.A.
Jump2Health TM[24]	USA	Taylor AL, 2016	Quasi-experimental	Parents	13	3-5 years	Nutrition, physical activity, screen time, sleep time	Website	The Jump2HealthTM Website provides statistics and research to highlight the importance of each healthy habit. The website also provides visitors with information and links on how to better meet expert recommendations for each healthy habit. These links include educational handouts, recipes, and cooking videos.	N.A.	N.A.
Jungle Gym[25]	USA	McCloskey M, 2018	Quasi-experimental	Children	24	3-5 years	Physical activity	App	The app featured animated characters on jungle adventures that encouraged movement. Children were instructed to move along with the characters.	Children tested the app once, lasting about 10 to 15 min per group.	N.A.

Lessonly (Baby-Act Trial)[26]	Puerto Rico	Kallis MG, 2023	Quasi-experimental	Parents	9	pregnancy - 1 year	Nutrition, physical activity, sleep	Website	The baby-act trial is a community-based educational intervention in partnership with the Women, Infants, and Children (WIC) program in Puerto Rico to prevent infant obesity. The website was developed as an alternative to a cell phone app. The website included 3 lessons that neede to be completed	N.A.	N.A.
Mini-KiSS Online[27]	Germany	Schlarb AA, 2012	Quasi-experimental	Parents	52 parents with 55 children	6 months - 4 years	Sleep	Website	The program consists of six treatment sessions designed to help parents implement sleep strategies gradually over six weeks. Each session includes educational content on sleep and behavioral strategies for parents, presented in a workbook format for recording behavior plans and summaries. To support the implementation of these strategies, parents receive additional materials, including a stuffed leopard for hypnotherapeutic techniques, short calming bedtime stories for observational learning, and audio recordings of imaginative exercises for parents to use with their children.	6 weeks	N.A.
MINISTOP[28]	Sweden	Nyström CD, 2017	RCT	Parents	315	4 years	Nutrition, physical activity (also mentiones sleep only twice)	App	App that delivers an extensive program of information to parents, divided into 12 themes (physical activity and sedentary behavior, candy and sweets, fruits and vegetables, drinks, eating between meals, fast food, sleep, foods outside the home, and foods at special occasions). Each theme consisted of general information, advice, and evidence-based strategies on how to change unhealthy behaviors. At the end of every week the parents received graphic feedback and automated comments based on the submitted information.	6 months	The control group received a pamphlet on healthy eating and physical activity in preschool-aged children based on the existing guidelines
MINISTOP[29]	Sweden	Nyström CD, 2018	RCT	Parents	263	4 years	Nutrition, physical activity	App	App that delivers an extensive program of information to parents, divided into 12 themes focused on children's health. Each theme consisted of general information, advice, and evidence-based strategies on how to change unhealthy behaviors. At the end of every week the parents received graphic feedback and automated comments based on the submitted information.	6 months	The control group received a pamphlet on healthy eating and physical activity in preschool-aged children based on the existing guidelines
MINISTOP 2.0[30]	Sweden	Alexandrou C, 2023	RCT	Parents	552	2·5–3 years	Nutrition, physical activity, screen time	App	The app provides parents with a comprehensive information program organized into 13 themes focused on children's health. In the 2.0 version, new features include audio and video content to enhance accessibility for users with varying literacy levels, as well as videos showcasing effective healthy eating strategies. Visual aids illustrate portion sizes, sweet intake limits, and fruit and vegetable recommendations for toddlers. The app also incorporates cultural adaptations, offering relatable recipes and reworked references to suit diverse backgrounds. Additionally, it includes images of children and families from various cultures.	6 months	The control group received the standard care offered by the Swedish primary child health care system during the routine visit. This includes a conversation about healthy foods and eating behaviors and health behaviors in general, as well as a pamphlet with information on healthy lifestyle behaviors.
N.A.[31]	USA	Lee H, 2023	RCT	Parents	73	1-3 years	Nutrition, physical activity, screen time	Website, SMS	The intervention was an 8-week eHealth program, combined with a provision of fresh fruit and vegetables biweekly. Weekly educational videos through the developed website and weekly reminder text messages with key information.	8 weeks	Participants in the control group received a booklet about general nutrition recommendations for children

									Parents were instructed to set parental feeding goals at the beginning of the intervention.		after completing baseline measurements.
NA[32]	USA	Sun A, 2017	RCT	Parents	8 (focus group) and 32 (RCT)	3-5 years	Nutrition, physical activity and screen time	Tablet-based educational tool	The intervention consisted of 8 weekly 30-minute, interactive, Cantonese, educational modules accessed online via tablet computers. The topics focus on healthy eating, grocery shopping, physical fitness, screen time reduction, and effective parenting strategies for maintaining a healthy weight.	8 weeks	Control group members received weekly mailings of printed health information relevant to preschool-aged children over the 8 weeks. The topics included an introduction to the study, food safety, choking hazards, oral health, immunizations, appropriate antibiotic use, injury prevention, and disaster preparation.
Nenne Navi[33]	Japan	Yoshizaki A, 2020	Quasi-experimental	Parents	10	1·5-3 years	Sleep	App	The e-learning content on sleep health was delivered via narrated animations, offering tips for daily routines that promote good sleep. Caregivers provided data on their children's sleep habits, which was analyzed by a pediatric sleep team of doctors and psychologists, who then delivered tailored advice to parents, who implemented a plan-do-check-act approach to improve sleep.	2 months	N.A.
Nenne Navi[34]	Japan	Yoshizaki A, 2023	Quasi-experimental	Parents	87	Age not specified, children included had a mean age of 19·5 months	Sleep	App	The e-learning content on sleep health was delivered via narrated animations, offering tips for daily routines that promote good sleep. Caregivers provided data on their children's sleep habits, which was analyzed by a pediatric sleep team of doctors and psychologists, who then delivered tailored advice to parents, who implemented a plan-do-check-act approach to improve sleep.	1 year	Video-only group, only receiving educational video content regarding sleep health literacy.
Sammen Happie![35]	The Netherlands	Karssen L, 2022	RCT	Parents	357	5-15 months	Nutrition, physical activity and sleep	App	The program was delivered via a stand-alone, easy-to-use app consisting of five age-based modules: 7–12, 12–15, 15–18, 18–24, and 24–28 months. Each age-based module provided parents with information (i.e., lessons) and exercises (i.e., challenges) about healthy parenting practices with respect to child EBRBs, as well as parental wellbeing and child temper (only lessons). By employing techniques that tackle (unhealthy) automatic behaviors, parents were encouraged to implement (newly learned) parenting skills as habits.	1 year	Parents that were allocated to the waitlist-control condition knew that they would receive access to the app at the end of the trial
Skoolbag/SWAP IT[36]	Australia	Pearson N, 2022	RCT	Parents	400	3-6 years	Nutrition	App	The SWAP IT for Childcare intervention comprised 3 components. 11 push notifications in 10 weeks. Each push notification alerted users to a within-app message, which aimed to target parent barriers to packing healthy lunch boxes. Several of the within-app messages provided a weblink to SWAP IT Options—a comprehensive list of foods suitable for packing in the lunch box. Links to other supporting information relevant to each message were also	10 weeks	The control group did not receive any intervention.

									provided, including fact sheets, short videos, and website links.		
Smart Moms[37]	USA	Nezami B, 2017	RCT	Parents	61	3-5 years	Nutrition	Website, SMS	The intervention focused on mothers as change agents to reduce their preschool-aged children's intake of sugar-sweetened beverages and juice, while also providing weight loss support for the mothers. It included one group session and a mobile-optimized website featuring 12 weekly lessons followed by six biweekly lessons. Mothers tracked their and their child's beverage consumption, daily red food intake, and weight, submitting this data via text message for tailored feedback from the intervention team. Additionally, they received motivational text messages and reminders 3-4 times a week.	6 months	Participants in the waitlist control group did not receive any intervention from baseline to 6 months but received a modified version of the intervention after 6-month assessments were complete.
Smart Moms[38]	USA	Nezami B, 2020	RCT	Parents	62	3-5 years	Nutrition	Website, SMS	The intervention focused on mothers as change agents to reduce their preschool-aged children's intake of sugar-sweetened beverages and juice, while also providing weight loss support for the mothers. It included one group session and a mobile-optimized website featuring 12 weekly lessons followed by six biweekly lessons. Mothers tracked their and their child's beverage consumption, daily red food intake, and weight, submitting this data via text message for tailored feedback from the intervention team. Additionally, they received motivational text messages and reminders 3-4 times a week.	6 months	Participants in the waitlist control group did not receive any intervention from baseline to 6 months but received a modified version of the intervention after 6-month assessments were complete.
Sugar Fact intervention[39]	Taiwan	Chen Y, 2020	Quasi-experimental	Parents	122	1-6 year	Nutrition	Youtube videos	The nutrition intervention included two 15 min online video sessions.	2x 15 minutes	For the plus group, the nutrition intervention included two 15 min online video sessions and one small-group discussion led by the researchers (for 2–3 h).
The Customized Sleep Profile (CSP)[40]	USA	Mindell JA, 2011	RCT	Parents	264	6-36 months	Sleep	Website, internet-based interventio n	The CSP is an algorithm-based internet intervention, including (1) a normative comparison of their child's sleep to other children of the same age; (2) a rating of whether their child is an “excellent, good, or disrupted sleeper”; (3) customized advice on how caregivers can help their child sleep better at night.	3 weeks	The mothers in the control group were instructed to follow their child’s usual bedtime practices throughout the entire 3-week period. They were informed that the study was about children’s bedtime activities and sleep behaviors.
The Customized Sleep Profile (CSP)[41]	USA	Mindell JA, 2011	RCT	Parents	171	6-36 months	Sleep	Website, internet-based interventio n	3 groups, internet, internet + routine and control. The CSP is an algorithm-based internet intervention. (1) a normative comparison of their child's sleep to other children of the same age; (2) a rating of whether their child is an “excellent, good, or disrupted sleeper”; (3) customized advice on how caregivers can help their child sleep better at night.	3 weeks	The mothers in the control group were instructed to follow their child’s usual bedtime practices throughout the entire 3-week period. They were informed that the study was about children’s

											bedtime activities and sleep behaviors.
The Short Messaging System (SmS) Parent Action Intervention[42]	USA	Brown B, 2019	Quasi-experimental	Parents	17	3-5 years	Nutrition, physical activity, screen time and sleep	SMS	The SmS Parent Action Intervention used text messaging to provide information on child nutrition, PA, sleep and screen time behaviours to parents of preschool children. The text messages were sent using MOsio, an online platform for test message automation that tracks whether participants received messages and responded to messages and their responses.	5 weeks	N.A.
Time2bHealthy[43]	Australia	Hammersley M, 2019	RCT	Parents	86	2-5 years	Nutrition, physical activity, screen time and sleep	Website, Facebook, e-mail	The intervention targeted multiple behaviors and consisted of 6 modules including an introduction, nutrition (n=2), physical activity, screen time, and sleep module. Each module comprised reading material, videos, activities, quizzes, and a goal-setting component. Each module took approximately 30 min to complete. Participants received weekly e-mails and feedback from the facilitator (a dietitian) on the goal-setting component. They were also encouraged to access and contribute to a closed Facebook group to communicate with other participants and the dietitian. allowing the opportunity to connect with others, gain a greater understanding of program content, pose questions to others, and share ideas and experiences. Post-program, participant received fortnightly e-mails until 6-month follow-up to recap on key information.	11 weeks (after which participants received e-mails until 6-month follow-up)	The comparison group received 11 weekly e-mails with links to information on similar topics on the evidence-based Raising Children Network website. Similar to the intervention group, during the maintenance period, they received fortnightly e-mails which revised the information sent in the previous e-mails.
Time2bHealthy[44]	Australia	Hammersley M, 2019	RCT	Parents	86	2-5 years	Nutrition, physical activity, screen time and sleep	Website, Facebook	The intervention targeted multiple behaviors and consisted of 6 modules including an introduction, nutrition (n=2), physical activity, screen time, and sleep module. Each module comprised reading material, videos, activities, quizzes, and a goal-setting component. Each module took approximately 30 min to complete. Participants received weekly e-mails and feedback from the facilitator (a dietitian) on the goal-setting component. They were also encouraged to access and contribute to a closed Facebook group to communicate with other participants and the dietitian. allowing the opportunity to connect with others, gain a greater understanding of program content, pose questions to others, and share ideas and experiences. Post-program, participant received fortnightly e-mails until 6-month follow-up to recap on key information.	11 weeks (after which participants received e-mails until 6-month follow-up)	The comparison group received 11 weekly e-mails with links to information on similar topics on the evidence-based Raising Children Network website. Similar to the intervention group, during the maintenance period, they received fortnightly e-mails which revised the information sent in the previous e-mails.
Time2bHealthy[45]	Australia	Hammersley M, 2020	Quasi-experimental evaluation within RCT	Parents	86	2-5 years	Nutrition, physical activity, screen time and sleep	Website, Facebook	The intervention targeted multiple behaviors and consisted of 6 modules including an introduction, nutrition (n=2), physical activity, screen time, and sleep module. Each module comprised reading material, videos, activities, quizzes, and a goal-setting component. Each module took approximately 30 min to complete. Participants received weekly e-mails and feedback from the facilitator (a dietitian) on the goal-setting component. They were also encouraged to access and contribute to a closed Facebook group to communicate with	11 weeks (after which participants received e-mails until 6-month follow-up)	N.A.

									other participants and the dietitian. allowing the opportunity to connect with others, gain a greater understanding of program content, pose questions to others, and share ideas and experiences. Post-program, participant received fortnightly e-mails until 6-month follow-up to recap on key information.		
Time2bHealthy[46]	Australia	Jones R, 2011	Quasi-experimental	Parents	47	2-5 years	Nutrition, physical activity and screen time	Website	The Time2bHealthy Program comprises five modules focusing on the key health areas related to unhealthy weight gain: dietary intake, physical activity and sedentary behaviours. Each module is completed over a two-week period. Each module contains personalised interactive activities, mediated discussion forums for parents and behaviour change components, such as goal setting. At the end of each module, parents are encouraged to write two SMART goals. The health consultant then liaises regularly, via personalised and group e-mails, with participants to ensure that their goals are accomplished and to minimise any potential barriers to completion. parents are able to ask the health consultant specific questions about the content of the program or their individual family circumstances	10 weeks	N.A.
Time2bHealthy, Healty habits plus[47]	Australia	Hammersley M, 2021	Partially Randomized Preference Trial	Parents	458	2-6 years	Nutrition, physical activity, screen time and sleep	Website, Facebook	The intervention comprised six modules focusing on healthy eating and movement behaviours, modules took 30 minutes to complete and included written content, practical activities, videos and goal setting were also encouraged to access and contribute to a closed Facebook group to communicate with other participants and the dietitian	12 weeks (up to 20 weeks to allow participants to complete the intervention)	The active control received written information (total of 10 information sheets and a summary booklet) on current recommendations for child healthy eating and movement behaviours. The Healthy Habits plus group received 20-30 min telephone support calls, focusing on healthy eating and movement behaviours.
Time2bHealthy, Healty habits plus[48]	Australia	Hammersley M, 2022	Partially Randomised Preference Trial	Parents	458	2-6 years	Nutrition, physical activity, screen time and sleep	Website, Facebook	At the start of the trial participants were asked if they had a strong preference for the way they received advice or support. If yes, then options were named; written information, telephone or online. In case of strong preference allocated to that intervention. If they did not express a strong preference, they were randomised to one of the three interventions (using a 1:1:1 ratio). Time2bHealthy (online) vs Healthy Habits plus (telephone) vs control, preference and randomised. Time2bHealthy online intervention, six modules focusing on healthy eating and movement behaviours, modules took 30 minutes and included written content, practical activities, videos and goal setting, also join Facebook group, 3 month period.	12 weeks (up to 20 weeks to allow participants to complete the intervention)	The active control received written information (total of 10 information sheets and a summary booklet) on current recommendations for child healthy eating and movement behaviours. The Healthy Habits plus group received 20-30 min telephone support calls, focusing on healthy eating and movement behaviours.

N.A.: Not Applicable
RCT: Randomized Controlled Trial
WIC: Woman, Infants and Children

Supplemental appendix 3. Definitions of outcomes in this scoping review

Table 1. Overview of definitions of outcomes used in this scoping review	
**Outcome**	**Definition**
Acceptability[49]	A multi-faceted construct, represented by at least one of seven components:<br>• How an individual feels about the intervention.<br>• The perceived amount of effort required to participate in the intervention.<br>• The extent to which the intervention fits with an individual's value system.<br>• The extent to which the participant understands the intervention and how it works<br>• The extent to which benefits, profits or values must be given up to engage in the intervention.<br>• The extent to which the intervention is perceived as likely to achieve its purpose.<br>• The participant's confidence that they can perform the behavior(s) required to participate in the intervention (self-efficacy).
Adherence[50]	Three elements are necessary to determine adherence to eHealth technology: (1) the ability to measure the usage behavior of individuals; (2) an operationalization of intended use; and (3) an empirical, theoretical, or rational justification of the intended use.
Adoption[51]	The absolute number, proportion, and representativeness of settings and intervention agents (people who deliver the program) who are willing to initiate a program, and why.
Attrition[52]	The number of participants who were lost to follow-up (e.g. participants do not return to fill in follow-up questionnaires).
Cost-effectiveness[53]	Used to determine the clinical benefit-to-cost ratio of a given intervention.
Dissemination[54]	The targeted distribution of information and intervention materials to a specific public health or clinical practice audience. The intent is to spread knowledge and the associated evidence-based interventions.
Effectiveness[51]	The impact of an intervention on the defined outcomes, including potential negative effects, and broader impact including quality of life and economic outcomes; and variability across subgroups (generalizability or heterogeneity of effects) in the real-world setting (effectiveness) or in the experimental/ideal setting (efficacy).
Engagement[55]	The affective, cognitive, and behavioral user experience. Affective engagement is related to emotions that people feel when seeing their progress, or a lack thereof, and related to emotions such as enjoyment felt when using the technology itself. Lastly, it entails identity: engaged users seem to identify themselves in some way with the technology or with the goal of the technology. Cognitive engagement is related to the technology being able to support and motivate people in reaching their goals, such as the goal of improving one's wellbeing. Moreover, it entails that engaged users are willing to spend mental effort in using the health intervention because it helps them achieve their goals, and they are intrinsically motivated. Engaged behavior includes the existence of a routine in which individuals use the technology, low effort required to use the technology, and technology usage that is not fixed but may fluctuate to fit with the needs of the current moment.
Implementation[51]	The intervention agents' fidelity to the various elements of an intervention's key functions or components, including consistency of delivery as intended and the time and cost of the intervention. Importantly, it also includes adaptations made to interventions and implementation strategies. Implementation is assessed by reporting on what percentage of process objectives were achieved (e.g., what proportion of pamphlets were distributed, how many class hours were taught, or prescribed phone calls completed). Further, very few studies report costs or specific staff time commitments associated with intervention implementation – information often very important for determining if others will attempt to try a program.
Operability[56]	The degree to which a product or system has attributes that make it easy to operate and control.
Usability[57]	Usability refers to the quality of a product or service which allows users to use it effectively and without effort, immediately learning its use and easily remembering it when returning to usage after a certain amount of time.
Usage[58]	The user's interactions with the intervention (e.g., duration, number of logins, number of days used, or exposure to intervention content).

Supplemental table 1. Outcome effectiveness

Table 1. Outcome Effectiveness (n=38)

Lifestyle component	Outcome measure	Method	Example question
Sleep	Child sleep parameters<br>• Bedtime<br>• Night-wakings<br>• Sleep onset<br>• Sleep duration<br>• Wake-up time	Actigraph GT3X+ accelerometers<br>Data entered in eHealth modality<br>Questionnaires<br>▪ BISQ(-R)<br>Sleep diary	How many times does your child usually wake during the night? *(BISQ-R)*
	Child sleep behavior<br>• Bedtime routine<br>• Sleep hygiene factors<br>• Bedtime difficulties	Questionnaires<br>• BRQ<br>• CBCL 1.5-5 (sleep items)<br>• CSHQ<br>Interviews	Does the child watch TV in bed?<br>Do you consider your Child's sleep as a problem?
	Parental outcomes<br>▪ Sleep quality<br>▪ Mood<br>▪ Behaviors	Questionnaires<br>▪ PSQI<br>▪ POMS<br>Interviews	What do you do if your child refuses to sleep?
Diet	Child dietary intake<br>▪ Complete<br>▪ Discretionary foods and beverages<br>▪ Fruit and vegetable<br>▪ Sugar-free or sweetened beverages<br>▪ Candy<br>▪ Mean energy provided by discretionary foods<br>▪ Mean energy, saturated fat, free sugar, and sodium of food in lunch box, provided and consumed<br>▪ Number of serves of core food groups*	Questionnaires<br>▪ FFQ<br>▪ CDQ<br>▪ EPAQ<br>▪ NSW CHS<br>▪ 24h-recall<br>Food diary<br>• Easy Diet Diary app<br>Pictures of child's plate<br>• TECH<br>Interviews<br>Weighed food records collected by researchers	Which of the following has been eaten over the past 7 days? *(CDQ)*
	Child eating behavior<br>▪ Willingness to try new food<br>▪ Eating a shared meal instead of eating separate meals<br>▪ Playing or watching tv during meals<br>▪ Sitting at the dinner table during meals<br>▪ Eating breakfast daily<br>▪ Drinking sweetened beverages	Questionnaire<br>• CEBQ<br>• CFNS<br>• FEAHQ	My child refuses new foods at first. (*Likert scale, CEBQ)*<br><br>How often does the child eat while watching television, reading or working?
	Child anthropometrics<br>• Weight status<br>• BMI	Questionnaire<br>• NSW CHS<br>Measured by healthcare professional	

	Parental outcomes<br>• Behaviors<br>• Feeding practices<br>• BMI	Questionnaire<br>• CFPQ<br>• CFQ (subscales restriction and pressure to eat)<br>• IFQ<br>• IFQS<br>• FEAHQ<br>Interviews	How many healthy meals did you make for your family last week?<br><br>How often do you or your spouse eat with the child? *(FEAHQ)*
Screen time	Child screen time parameters<br>• Amount of screen time<br>• Days with electronic media devices use for less than 1 hour	Questionnaire<br>• the National Nutrition and Physical Activity Survey.<br>• EY-PAQ	How many days each week and for how long each day has your child spent watching TV?^ *(EY-PAQ)*
	Parental outcomes<br>• Changes made or attempted<br>• Household Televison use	Interview<br>Questionnaire	
Physical activity	Child physical activity parameters<br>• Duration of physical activity<br>• Time spent in MVPA<br>• Days with at least 3 hours of activity	Questionnaire<br>• the National Nutrition and Physical Activity Survey.<br>• EY-PAQ<br>Actigraph GT3X+ accelerometers	On how many days each week and for how long each day has your child played at the park/playground?^ *(EY-PAQ)*

^ Shortened version of the question
* Core food groups: bread and cereals, fruits, vegetables, dairy, meat, and meat alternatives
BISQ(-R): Brief Infant Sleep Questionnaire (Revised)
BRQ: Bedtime Routines Questionnaire
CDQ: Children's Dietary Questionnaire
CFPQ: Comprehensive Feeding Practices Questionnaire
CFQ: Child Feeding Questionnaire
CSHQ: Children's sleep habits questionnaire
EPAQ: Eating and Physical Activity Questionnaire
EY-PAQ: The Early Years Physical Activity Questionnaire
IFQ: Infant Feeding Questionnaire
IFQS: Infant Feeding Style Questionnaire
MVPA: Moderate or Vigorous Physical Activity
NSW CHS: New South Wales Child Population Health Survey
PSQI: Pittsburgh Sleep Quality Index
POMS: Profile of Moods States
TECH: Tool for Energy Balance in Children

Supplemental table 2. Outcome acceptability

Table 2. Outcome acceptability^ (n=41)

**Outcome measure**	**Example question**
User's feelings towards the intervention (content, features, characteristics and participation)<br>• Relevance<br>• Appropriateness<br>• Comprehensiveness<br>• Interestingness<br>• Amount of information<br>• Perceived correctness<br>• Literacy level<br>• Communication style<br>• Specific feature<br>• Interface<br>• Layout<br>• Dosage<br>• Timing<br>• Mode of delivery<br>• Format<br>• Participating in the intervention<br>• Likelihood of participating again in similar intervention<br>• Suggestions for improvement<br>• Likelihood of paying for the intervention<br>• Likelihood of continued use<br>• Sharing intervention content with others<br>• Likelihood of recommendation to others	• The intervention answered nearly all my questions. *(Likert scale)*<br>• The content was adapted to the child's age. *(Likert scale)*<br>• The program was easy to understand. *(Likert scale)*<br>• I was interested in the parents' forum. *(Likert scale)*<br>• Would you have preferred to receive the intervention in another way?<br>• I am glad that I participated *(Likert scale)*<br>• How could the website be improved?<br>• I would recommend the intervention to other families *(Likert scale)*<br>• I am interested in continuing to use the app. *(Likert scale)*
Perceived intervention effectiveness<br>• Intervention in general<br>• Intervention recommendations<br>• Feature found most useful<br>• Perception of learning something new	• How helpful were the recommendations provided to you last year?<br>• The tips helped me give my child water instead of sugary drinks. (*Likert scale)*
Perceived amount of effort required to participate in the intervention<br>• Attrition rate<br>• Reasons for drop-out<br>• Intervention duration	• Why did you delete the intervention from your device?<br>• The length of the program was appropriate *(Likert scale)*
The participant's confidence that they can perform the behavior(s) required to participate in the intervention (self-efficacy).	• How certain are you that you can get your child to eat at least one fruit every day? Based on PSE questionnaire

^ Organized based on the Theoretical Framework of Acceptability[49].

Supplemental table 3. Outcome usage

Table 4. Outcome Usage (n=22)

Modality	Outcome measure	Method
Website	Number of users visiting the website	
	Parental self-reported use<br>• Frequency of website access<br>• Frequency viewing specific content<br>• Active engagement, interaction, and applied intervention materials<br>• Provided advice used	Questionnaires<br>Interviews
	Parental logged use<br>• Frequency of website visits<br>• Number of users of the website<br>• Frequency viewing specific content<br>• Completed intervention components<br>• Time spent viewing the website<br>• Activity on discussion forum	Website usage log<br>• Google Analytics<br>• From the website itself<br>• Learning Managment System NEO<br>Measurement at participating centre level
Facebook	Parental self-reported use<br>• Rate of participant activity	Questionnaires<br>Interviews
	Parental logged use<br>• Number of shared posts<br>• Watching Facebook live<br>• Watching videos<br>• Number of comments<br>• Number of likes<br>• Frequency of viewing specific content<br>• Number of posts posted by participant<br>• Sustained usage: interaction with last post of the intervention	Counted by researcher<br>Sociograph (a Facebook analytic tool)<br>Questionnaire<br>Facebook group content was reviewed
App	Parental self-reported use<br>• Overall use<br>• Using specific features<br>• Features or content that were not used or viewed<br>• Reasons for nonuse	Questionnaires<br>Interviews
	Parental logged use<br>• Entering requested data<br>• Number of logins<br>• Number of feedback messages read<br>• Frequency of overall usage<br>• Duration of overall usage<br>• Usage of a specific feature within the intervention	App usage log (monitoring website)<br>Points earned (each activity results in points the participant earns)<br>Monitoring reports<br>App use collected in online database
	Child's logged use<br>• Time spent engaged as directed<br>• Time spent engaged not as directed<br>• Time spent being distracted	Notes made during app interaction<br>Watching videotapes of app interaction
SMS	Parental logged use<br>• Response to program questions<br>• Completion of questionnaires<br>• Number of self-monitoring texts	Active participants: completion of pre- and post-survey and answering at least one program question<br>Recorded by Mosio (text message program)

References

1. Mindell JA, Leichman ES, Walters R, Bhullar B. Development and dissemination of a consumer health information website on infant and toddler sleep. *Transl Behav Med* 2021; **11**(9): 1699-707.
2. Kitsaras G, Pretty IA, Allan J. Bedtime Routines Intervention for Children (BRIC) project: results from a non-randomised feasibility, proof-of concept study. *Pilot Feasibility Stud* 2022; **8**(1): 79.
3. Hull P, Emerson JS, Quirk ME, et al. A Smartphone App for Families With Preschool-Aged Children in a Public Nutrition Program: Prototype Development and Beta-Testing. *Jmir Mhealth and Uhealth* 2017; **5**(8).
4. Zhang Q, Panichelli J, Hall LA. Assessment of Cooking Matters Facebook Platform to Promote Healthy Eating Behaviors among Low-Income Caregivers of Young Children in the United States: A Pilot Study. *Nutrients* 2021; **13**(8).
5. Garvin TM, Chiappone A, Boyd L, et al. Cooking Matters Mobile Application: a meal planning and preparation tool for low-income parents. *Public Health Nutr* 2019; **22**(12): 2220-7.
6. Ezran M, Trude ACB, Hepworth AD, Black MM. Parent Website Engagement and Health Equity Implications in a Child Care-Based Wellness Intervention. *Journal of Nutrition Education and Behavior* 2021; **53**(8): 654-62.
7. Helle C, Hillesund ER, Wills AK, Overby NC. Examining the effects of an eHealth intervention from infant age 6 to 12 months on child eating behaviors and maternal feeding practices one year after cessation: The Norwegian randomized controlled trial Early Food for Future Health. *PLoS One* 2019; **14**(8): e0220437.
8. Helle C, Hillesund ER, Wills AK, Overby NC. Evaluation of an eHealth intervention aiming to promote healthy food habits from infancy -the Norwegian randomized controlled trial Early Food for Future Health. *Int J Behav Nutr Phys Act* 2019; **16**(1): 1.
9. Knowlden AP, Sharma M, Cottrell RR, Wilson BR, Johnson ML. Impact evaluation of Enabling Mothers to Prevent Pediatric Obesity through Web-Based Education and Reciprocal Determinism (EMPOWER) Randomized Control Trial. *Health Educ Behav* 2015; **42**(2): 171-84.
10. Knowlden AP, Sharma M. Process evaluation of the Enabling Mothers toPrevent Pediatric Obesity Through Web-Based Learning and Reciprocal Determinism (EMPOWER) randomized control trial. *Health Promot Pract* 2014; **15**(5): 685-94.
11. Knowlden A, Sharma M. One-Year Efficacy Testing of Enabling Mothers to Prevent Pediatric Obesity Through Web-Based Education and Reciprocal Determinism (EMPOWER) Randomized Control Trial. *Health Educ Behav* 2016; **43**(1): 94-106.
12. Knowlden AP, Conrad E. Two-Year Outcomes of the Enabling Mothers to Prevent Pediatric Obesity Through Web-Based Education and Reciprocal Determinism (EMPOWER) Randomized Control Trial. *Health Educ Behav* 2018; **45**(2): 262-76.
13. Afonso L, Rodrigues R, Reis E, et al. Fammeal: A Gamified Mobile Application for Parents and Children to Help Healthcare Centers Treat Childhood Obesity. *Ieee T Games* 2020; **12**(4): 351-60.
14. Afonso L, Rodrigues R, Castro J, et al. A Mobile-Based Tailored Recommendation System for Parents of Children with Overweight or Obesity: A New Tool for Health Care Centers. *Eur J Invest Health* 2020; **10**(3): 779-94.
15. Roed M, Vik FN, Hillesund ER, Van Lippevelde W, Medin AC, Overby NC. Process Evaluation of an eHealth Intervention (Food4toddlers) to Improve Toddlers' Diet: Randomized Controlled Trial. *JMIR Hum Factors* 2020; **7**(3): e18171.
16. Roed M, Medin AC, Vik FN, et al. Effect of a Parent-Focused eHealth Intervention on Children's Fruit, Vegetable, and Discretionary Food Intake (Food4toddlers): Randomized Controlled Trial. *J Med Internet Res* 2021; **23**(2): e18311.
17. Fiks AG, Gruver RS, Bishop-Gilyard CT, et al. A Social Media Peer Group for Mothers To Prevent Obesity from Infancy: The Grow2Gether Randomized Trial. *Child Obes* 2017; **13**(5): 356-68.

18. Gruver RS, Bishop-Gilyard CT, Lieberman A, et al. A Social Media Peer Group Intervention for Mothers to Prevent Obesity and Promote Healthy Growth from Infancy: Development and Pilot Trial. *Jmir Res Protoc* 2016; **5**(3): 256-70.
19. Lawton K, Hess L, McCarthy H, Marini M, McNitt K, Savage JS. Feasibility of Using Facebook to Engage SNAP-Ed Eligible Parents and Provide Education on Eating Well on a Budget. *Int J Environ Res Public Health* 2022; **19**(3).
20. Evans MG, Fleckman J, Williams TT, Tokarz SM, Theall KP. Delivering Health Information to Parents via a Theory-Informed SMS-Based Intervention: Development and Results from a Pilot Study. *Matern Child Health J* 2022; **26**(1): 49-57.
21. Reyes LI, Johnson SL, Chamberlin B, Bellows LL. Engaging Preschoolers in Food Tasting and Movement Activities Using Mobile Applications. *J Nutr Educ Behav* 2023; **55**(1): 77-80.
22. Davies MA, Terhorst L, Nakonechny AJ, Skukla N, El Saadawi G. The development and effectiveness of a health information website designed to improve parents' self-efficacy in managing risk for obesity in preschoolers. *J Spec Pediatr Nurs* 2014; **19**(4): 316-30.
23. Leichman ES, Gould RA, Williamson AA, Walters RM, Mindell JA. Effectiveness of an mHealth Intervention for Infant Sleep Disturbances. *Behav Ther* 2020; **51**(4): 548-58.
24. Taylor AL, Reed DB, Colwell MJ. Qualitative Evaluation of the Jump2Health Website for Parents of Preschool Children Shows Behavior Changes. *Int Q Community Health Educ* 2016; **37**(1): 43-50.
25. McCloskey ML, Thompson DA, Chamberlin B, Clark L, Johnson SL, Bellows LL. Mobile Device Use Among Rural, Low-Income Families and the Feasibility of an App to Encourage Preschoolers' Physical Activity: Qualitative Study. *Jmir Pediatrics and Parenting* 2018; **1**(2).
26. Kallis MG, Campos M, Helen-Mays M, Palacios C. Acceptability and Ease of Use of an Educational Website among Women, Infants, and Children Program Participants in Puerto Rico: A Pilot Study. *P R Health Sci J* 2023; **42**(3): 246-8.
27. Schlarb AA, Brandhorst I. Mini-KiSS Online: an Internet-based intervention program for parents of young children with sleep problems - influence on parental behavior and children's sleep. *Nat Sci Sleep* 2012; **4**: 41-52.
28. Nystrom CD, Sandin S, Henriksson P, et al. Mobile-based intervention intended to stop obesity in preschool-aged children: the MINISTOP randomized controlled trial. *Am J Clin Nutr* 2017; **105**(6): 1327-35.
29. Delisle Nystrom C, Sandin S, Henriksson P, Henriksson H, Maddison R, Lof M. A 12-month follow-up of a mobile-based (mHealth) obesity prevention intervention in pre-school children: the MINISTOP randomized controlled trial. *BMC Public Health* 2018; **18**(1): 658.
30. Alexandrou C, Henriksson H, Henstrom M, et al. Effectiveness of a Smartphone App (MINISTOP 2.0) integrated in primary child health care to promote healthy diet and physical activity behaviors and prevent obesity in preschool-aged children: randomized controlled trial. *Int J Behav Nutr Phys Act* 2023; **20**(1): 22.
31. Lee H, Oldewage-Theron W, Dawson JA. Effects of a Theory-Based, Multicomponent eHealth Intervention for Obesity Prevention in Young Children from Low-Income Families: A Pilot Randomized Controlled Study. *Nutrients* 2023; **15**(10).
32. Sun A, Cheng J, Bui Q, Liang Y, Ng T, Chen JL. Home-Based and Technology-Centered Childhood Obesity Prevention for Chinese Mothers With Preschool-Aged Children. *J Transcult Nurs* 2017; **28**(6): 616-24.
33. Yoshizaki A, Mohri I, Yamamoto T, et al. An Interactive Smartphone App, Nenne Navi, for Improving Children's Sleep: Pilot Usability Study. *JMIR Pediatr Parent* 2020; **3**(2): e22102.
34. Yoshizaki A, Murata E, Yamamoto T, et al. Improving Children's Sleep Habits Using an Interactive Smartphone App: Community-Based Intervention Study. *JMIR Mhealth Uhealth* 2023; **11**: e40836.
35. Karssen LT, Larsen JK, Burk WJ, et al. Process and effect evaluation of the app-based parenting program

on infant zBMI: A randomized controlled trial. *Front Public Health* 2022; **10**.
36. Pearson N, Finch M, Sutherland R, et al. An mHealth Intervention to Reduce the Packing of Discretionary Foods in Children's Lunch Boxes in Early Childhood Education and Care Services: Cluster Randomized Controlled Trial. *Journal of Medical Internet Research* 2022; **24**(3).
37. Nezami BT, Ward DS, Lytle LA, Ennett ST, Tate DF. A mHealth randomized controlled trial to reduce sugar-sweetened beverage intake in preschool-aged children. *Pediatr Obes* 2018; **13**(11): 668-76.
38. Nezami BT, Lytle LA, Ward DS, Ennett ST, Tate DF. Effect of the Smart Moms intervention on targeted mediators of change in child sugar-sweetened beverage intake. *Public Health* 2020; **182**: 193-8.
39. Chen YC, Huang YL, Chien YW, Chen MC. The Effect of an Online Sugar Fact Intervention: Change of Mothers with Young Children. *Nutrients* 2020; **12**(6).
40. Mindell JA, Du Mond CE, Sadeh A, Telofski LS, Kulkarni N, Gunn E. Efficacy of an internet-based intervention for infant and toddler sleep disturbances. *Sleep* 2011; **34**(4): 451-8.
41. Mindell JA, Du Mond CE, Sadeh A, Telofski LS, Kulkarni N, Gunn E. Long-term efficacy of an internet-based intervention for infant and toddler sleep disturbances: one year follow-up. *J Clin Sleep Med* 2011; **7**(5): 507-11.
42. Brown B, Harris K, Dybdal L, Malich J, Bodnar B, Hall E. Feasibility of text messaging to promote child health in a rural community on an American Indian reservation. *Health Educ J* 2019; **78**(5): 557-69.
43. Hammersley ML, Okely AD, Batterham MJ, Jones RA. Investigating the mediators and moderators of child body mass index change in the Time2bHealthy childhood obesity prevention program for parents of preschool-aged children. *Public Health* 2019; **173**: 50-7.
44. Hammersley ML, Okely AD, Batterham MJ, Jones RA. An Internet-Based Childhood Obesity Prevention Program (Time2bHealthy) for Parents of Preschool-Aged Children: Randomized Controlled Trial. *J Med Internet Res* 2019; **21**(2): e11964.
45. Hammersley ML, Okely AD, Batterham MJ, Jones RA. Can Parental Engagement in Social Media Enhance Outcomes of an Online Healthy Lifestyle Program for Preschool-Aged Children? *Health Commun* 2020; **35**(9): 1162-71.
46. Jones R, Wells M, Okely A, Lockyer L, Walton K. Is an online healthy lifestyles program acceptable for parents of preschool children? *Nutr Diet* 2011; **68**(2): 149-54.
47. Hammersley ML, Wyse RJ, Jones RA, et al. Translation of Two Healthy Eating and Active Living Support Programs for Parents of 2-6-Year-Old Children: Outcomes of the 'Time for Healthy Habits' Parallel Partially Randomised Preference Trial. *Nutrients* 2021; **13**(10).
48. Hammersley ML, Wyse RJ, Jones RA, et al. Telephone and Web-Based Delivery of Healthy Eating and Active Living Interventions for Parents of Children Aged 2 to 6 Years: Mixed Methods Process Evaluation of the Time for Healthy Habits Translation Trial. *J Med Internet Res* 2022; **24**(5): e35771.
49. Sekhon M, Cartwright M, Francis JJ. Acceptability of healthcare interventions: an overview of reviews and development of a theoretical framework. *Bmc Health Serv Res* 2017; **17**.
50. Sieverink F, Kelders SM, van Gemert-Pijnen JE. Clarifying the Concept of Adherence to eHealth Technology: Systematic Review on When Usage Becomes Adherence. *J Med Internet Res* 2017; **19**(12): e402.
51. Glasgow RE, Harden SM, Gaglio B, et al. RE-AIM Planning and Evaluation Framework: Adapting to New Science and Practice With a 20-Year Review. *Front Public Health* 2019; **7**: 64.
52. Eysenbach G. The Law of Attrition. *Journal of Medical Internet Research* 2005; **7**(1).
53. Shi CR, Nambudiri VE. Research Techniques Made Simple: Cost-Effectiveness Analysis. *J Invest Dermatol* 2017; **137**(7): E143-E7.
54. Glasgow RE, Vinson C, Chambers D, Khoury MJ, Kaplan RM, Hunter C. National Institutes of Health Approaches to Dissemination and Implementation Science: Current and Future Directions. *American Journal of Public Health* 2012; **102**(7): 1274-81.
55. Kelders SM, Kip H, Greeff J. Psychometric Evaluation of the TWente Engagement with Ehealth Technologies Scale (TWEETS): Evaluation Study. *Journal of Medical Internet Research* 2020; **22**(10).

56. Ronchieri E, Canaparo M. Assessing the impact of software quality models in healthcare software systems. *Health Syst* 2023; **12**(1): 85-97.
57. Kip H, Beerlage-de Jong, N., van Gemert-Pijnen, J.E., Sanderman, R., Kelders, S.M. eHealth Research, Theory and Development: A Multi-Disciplinary Approach. . London: Routledge; 2018.
58. Siezenga A.M. MECA, van Gelder J-L. A look under the hood: analyzing engagement and usage data of a smartphone-based intervention. *BMC Digital Health* 2023; **1**(1).
